# A Novel Circular RNA hsa_circRPPH1_015 Exerts an Oncogenic Role in Breast Cancer by Impairing miRNA-326-Mediated ELK1 Inhibition

**DOI:** 10.3389/fonc.2020.00906

**Published:** 2020-06-24

**Authors:** Chunming Zhao, Linlin Li, Zhiwei Li, Jiawen Xu, Qing Yang, Peng Shi, Kaining Zhang, Rui Jiang

**Affiliations:** ^1^Department of Geriatrics, Shandong Provincial Hospital Affiliated to Shandong First Medical University, Jinan, China; ^2^Department of Oncology, Shandong Provincial Hospital Affiliated to Shandong First Medical University, Jinan, China; ^3^Department of Thyroid and Breast Surgery, Shandong Provincial Hospital Affiliated to Shandong First Medical University, Jinan, China; ^4^Department of Pathology, Shandong Provincial Hospital Affiliated to Shandong First Medical University, Jinan, China; ^5^Department of Ultrasound, Shandong Provincial Hospital Affiliated to Shandong First Medical University, Jinan, China

**Keywords:** breast cancer, Circular RNA, hsa_circRPPH1_015, microRNA-326, ELK1

## Abstract

**Background:** Breast cancer (BC) represents a heterogeneous disease with distinct subtypes and high tumor metastatic potentials. Recent researchers identify the implication of circular RNAs (circRNAs) in the initiation of BC. Herein, we uncover a novel circRNA hsa_circRPPH1_015 as a tumor promoter in BC.

**Methods:** A total of 86 paired cancerous and non-cancerous tissues were surgically resected and collected from BC patients. Cell proliferation, colony formation, and cell invasion were examined by Edu staining, clone formation assays, propidium iodide (PI)-labeled flow cytometry, and Transwell invasion assays. PCNA, Ki67, MMP-2, MMP-9, Cyclin D1, and CDK4 expression was assayed using Western blot analysis. RNA pull-down, dual-luciferase reporter gene assay, and RNA binding protein immunoprecipitation (RIP) assay were performed to investigate the relationships among hsa_circRPPH1_015, microRNA-326 (miR-326), and ELK1. The tumor growth of human BC MCF-7 cells *in vivo* was evaluated in nude mice by subcutaneous xenografts of MCF-7 cells.

**Results:** hsa_circRPPH1_015 expression was upregulated in BC tissues. Knockdown of hsa_circRPPH1_015 restrained the aggressive behavior of MCF-7. hsa_circRPPH1_015 could bind to miRNA-326 that negatively regulates ELK1. Elevation of miRNA-326 expression resulted in inhibition of cell proliferation, colony formation, and cell invasion of MCF-7. Disturbance of miRNA-326 or overexpression of ELK1 restored the proliferation and aggressiveness in hsa_circRPPH1_015-depleted MCF-7 cells. Tumor growth of MCF-7 cells *in vivo* was reduced in nude mice lack of endogenous hsa_circRPPH1_015 expression.

**Conclusion:** Overall, the present study demonstrates that hsa_circRPPH1_015 was an oncogene and unfavorable prognostic factor in BC, providing an exquisite therapeutic target for BC.

## Background

Breast cancer (BC) is recognized as a complex cancer at an increasing prevalence among all types of cancers within the female population (1). Statistics showed about 12.2% of all newly diagnosed BC cases in China and 9.6% of all BC-induced deaths on a global scale (2). Typically, three distinct subtypes of BC have been identified, where their therapeutic strategies can be developed depending on the presence of molecular markers for progesterone or estrogen receptors and human epidermal growth factor 2 (3). In spite of the improved understanding of BC, the novel molecular-targeted treatment modalities are urgently needed to combat the disease (4).

In recent years, circular RNAs (circRNAs) have attracted growing attention in research regarding their functional role and their interplay with microRNAs (miRNAs) in cancers (5). For instance, hsa_circ_0001982 has been reported to regulate BC cell biological functions by decreased expression of microRNA-143 (miR-143) (6). Besides, the overexpression of hsa_circ_0052112 has been indicated to promote the cell migration and invasion of BC by binding to miR-125a-5p in Zhang et al. (7). miRNAs are short, non-coding RNAs that function as key post-transcriptional regulators (8). The available evidence has been proposed that miRNAs as the promising therapeutic targets for diseases (9). Prior to the current investigation, a targeting relationship between miR-326 and circRNA hsa_circ_0000517 (hsa_circRPPH1_015) was assessed by using CircInteractome database (https://circinteractome.nia.nih.gov/index.html). miR-326 highlights itself as an antioncomiR in pro-static carcinoma by negatively regulates Mucin1 (10). Additionally, miR-326 has been noted to harbor tumor-suppressive property in BC (11). Furthermore, the TargetScan database revealed that there were binding sites between miR-326 and ETS-domain containing protein (ELK1). The literature on the involvement of ELK1 in cancers is burgeoning. For instance, the overexpression of ELK1 has been reported to relate to dismal recurrence-free survival in patients with triple-negative BC (12). As a transcription factor, ELK1 has been verified to induce the up-regulation of BC cell proliferation modules (13). ELK1 and ELK3 have been identified as the target genes of miR-135a that play a suppressive role in BC (14). Importantly, the circRNA-miRNA-mRNA axis has been found to be implicated in BC (15, 16). Based on the aforementioned research, this study was initiated with the purpose to explore the regulatory mechanism of circRNA hsa_circRPPH1_015/miR-326/ELK1 in BC which may provide a novel biomarker to enhance the clinical efficacy of BC treatment.

## Materials and Methods

### Ethics Statement

This study was approved by the Ethics Committee of Shandong Provincial Hospital Affiliated to Shandong First Medical University and conformed to the Helsinki Declaration. All patients signed informed consent forms. The animal experiments were performed with the approval of the Animal Ethics Committee.

### Patient Enrollment

The cancerous tissues of 86 BC patients above 18 years old (mean age: 55.02 ± 12.03 years) were collected in Shandong Provincial Hospital Affiliated to Shandong First Medical University from January 2012 to December 2013 (17). The adjacent normal tissues located 5 cm away from the corresponding cancer tissues were collected as the control. There were 40 patients with tumor diameter <2 cm, 30 patients with tumor diameter from 2 to 5 cm, and 16 patients with tumor diameter > 5 cm. There were 44 patients at stage I, 27 patients at stage II, and 15 patients at stage III. There were 40 patients at grade I, 25 patients at grade II, and 21 patients at grade III. A total of 86 patients were followed for 5 years while 27 patients passed away during the follow-up period.

### Cell Treatment

BC cell lines, MCF-7, MDA-MB-435, MDA-MB-231, BCAP, and human normal breast cell line HBL-100 were purchased from American Type Culture Collection (ATCC; Manassas, VA, USA). The cells were cultured in Roswell Park Memorial Institute (RPMI)-1640 medium containing 10% fetal bovine serum (FBS), 100 μg/mL streptomycin, and 100 U/mL penicillin, with 5% CO_2_ at 37°C. When the confluence of cells reached about 90%, the cells were treated with 0.25% trypsin to the cell circle, which was terminated by adding the culture medium. Then the cells are blown into a uniform single cell suspension. Reverse transcription quantitative polymerase chain reaction (RT-qPCR) was used to detect the expression of hsa_circRPPH1_015 in cell lines, and the cell line with the highest hsa_circRPPH1_015 expression was selected for subsequent experiments.

Next, the cells at the logarithmic growth phase were transfected with plasmids of small interfering RNA (si)-circ-hsa_circRPPH1_015, miR-326 mimic, miR-326 inhibitor, si-circ_hsa_circRPPH1_015 + miR-326 inhibitor, overexpression (oe)-ELK1, si-circ_hsa_circRPPH1_015 + oe-ELK1, and their corresponding controls [si-negative control (NC), mimic NC, inhibitor NC, oe-NC]. All plasmids were purchased from Shanghai Genechem Co., Ltd., (Shanghai, China).

Cells were inoculated into 6-well-plates at a density of 3 × 10^5^ cells/well. Upon reaching about 50% confluence, cells were transfected using Lipofectamine 2000 (11668019, Invitrogen, Carlsbad, California, USA). In brief, 4 μg target plasmids and 10 μL Lipofectamine 2000 were diluted in 250 μL serum-free Opti-minimum essential medium (MEM) (51985042, Gibco, Carlsbad, California, USA), and then mixed by gentle shaking, followed by incubation at room temperature for 5 min. Next, the two solution was mixed, and allowed to stand for 20 min, which was added to each well. The plate was gently mixed and cells were further cultured in a 5% CO_2_ incubator at 37°C. With the medium renewed after 6 h of transfection, the cells were cultured for additional 36–48 h and then harvested for subsequent experiments.

### Northern Blot Analysis

The total RNA in tissues was extracted, separated by sodium dodecyl sulfate polyacrylamide gel electrophoresis (SDS-PAGE) for 2 h, transferred from denatured gel onto nitrocellulose membrane, and baked at 80°C for 2 h. Next, the membrane was prehybridized with 5 mL of prehybridization solution at 42°C for 3 h, and then hybridized with hybridization solution containing digoxin-labeled probe (primer sequences are listed in **Table 2**) at 42°C for 16 h. The next day, the membrane was washed with 2 × SSC/0.1%SDS (20 × SSC: 175.3 g NaCl, 88.2 g trisodium citrate; added to 800 mL by ddH_2_O, pH adjusted to 7.0 by 2N NaOH, and finally added to 1,000 mL by ddH_2_O). Next, the probe was detected with anti-digoxin antibody and Fab fragments.

### RT-qPCR

Primers were designed on both sides of the specific back splicing site of circRNA to effectively distinguish circRNA and linear RNA (Figure S1A). The total RNA in tissues and cells was extracted using Trizol (Invitrogen, USA). The complementary DNA (cDNA) was generated using Taqman MicroRNA Assays (Applied Biosystems, USA). Briefly, the non-RNase Eppendorf (EP) tube was added with 7 μL reverse transcription solution [0.15 μL 100 mM dNTPs (with dTTP), 1.00 μL 50 U/μL Reverse Transcriptase, 1.50 μL 10 × Reverse Transcription Buffer, 0.19 μL 20 U/μL RNase Inhibitor, and 4.16 μL Nuclease-free Water], 5 μL total RNA, and 3 μL 5 × RT primer at 16°C for 30 min, 42°C for 30 min, and 85°C for 5 min. The primers (Table 1) were synthesized by Wuhan Sangon Biotech Co., Ltd., (Wuhan, Hubei, China). Real-time fluorescence qPCR was performed using ABI7500 qPCR instrument (7500, ABI Company, Oyster Bay, NY, USA). The relative expression levels of genes were calculated by 2^−ΔΔCt^ method. U6 was used for miR-326 normalization and glyceraldehyde-3-phosphate dehydrogenase (GAPDH) for mRNA normalization.

**Table 1:** Primer sequences for RT-qPCR.

**Genes**	**Primer sequences (5^**′**^-3^**′**^)**
hsa_circRPPH1_015	F: AGCTTGGAACAGACTCACGG
	R: AATGGGCGGAGGAGAGTAGT
hsa_circRPPH1_015 FAM labeled probe	F: CGGCCCTAACAGGGCTCTCC
	R: GCCGGGCCCCTCCCCGAAGG
miR-326	F: GGCGCCCAGAUAAUGCG
	R: CGTGCAGGGTCCGAGGTC
ELK1	F: AGTCAAAGTGGAGGGGCCTA
	R: TCCTGATCCTGGAGTCCGTT
U6	F: TGTTCCACACTACGCAGTCC
	R: TTTGTCGTTCCCGTCTCCTG
GAPDH	F: GGAGCGAGATCCCTCCAAAAT
	R: GGCTGTTGTCATACTTCTCATGG
PALM	F: CCACAGCCTCACGGGTCCAG
	R: GGGGGTCCTTGTCTCGGGAA
KCNIP2	F: TCTCCTGCTATGGTGCTTCC
	R: TATCCATTGACTCAGGCCCA
VLDLR	F: ACTTTTTTTTCATGGGAGGT
	R: GAATGCTAACCTACTTACC
LRRTM1	F: TCTCTGTCTATACTGGCTGC
	R: GCGCCTCGGTGAGGTTGAGC

*RT-qPCR, reverse transcription quantitative polymerase chain reaction; ELK1, ETS-domain containing protein; GAPDH, glyceraldehyde-3-phosphate dehydrogenase; KCNIP2, Kv (potassium) channel interacting protein 2; VLDLR, very low-density lipoprotein receptor; LRRTM1, leucine rich repeat transmembrane neuronal 1; F, forward; R, reverse*.

### Western Blot Analysis

Radio-Immunoprecipitation lysis (BB-3209, BestBio, Shanghai, China) was used to isolate the total protein from the cells. The protein was separated by SDS-PAGE and transferred onto the polyvinylidene fluoride membrane. The membrane was allowed to probe with primary antibodies including rabbit monoclonal antibodies to proliferating cell nuclear antigen (PCNA) (1:1,000, ab92552, Abcam Inc., Cambridge, UK), Ki-67 (1:5,000, ab92742, Abcam Inc.), matrix metalloproteinase (MMP)-2 (1:1,000, ab92536, Abcam Inc.), MMP-9 (1:1,000, ab38898, Abcam Inc.), Cyclin D1 (1 : 200, ab16663, Abcam Inc.), and Cyclin-dependent kinase 4 (CDK4) (1:1,000, ab108357, Abcam Inc.). Horseradish peroxidase-labeled goat anti-rabbit IgG secondary antibody (1:1,000, Wuhan Boster Biological Technology Co., Ltd., Wuhan, Hubei, China) was added and incubated with the membrane. Target protein relative expression = gray value of the target protein band/gray value of GAPDH.

### 5-ethynyl-2′-deoxyuridine (EdU) Assay

EdU solution was added to the cell culture plate and incubated for 2 h at room temperature. The cells were then fixed with 40 g/L paraformaldehyde for 30 min, incubated with glycine solution for 8 min and then washed with phosphate-buffered saline (PBS) containing 0.5% Triton X-100. After that, Apollo® staining reaction solution was added and incubated for 30 min at room temperature in the dark. Methanol and PBS were then used to wash the cells 2 times, respectively. Finally, the cells were incubated with Hoechst 33342 reaction solution (C1022, Beyotime Biotechnology Co., Shanghai, China) at room temperature for 20 min in the dark. Under the fluorescence microscope, the red-stained cells (EdU-positive) were the proliferating cells and the blue-stained cells (Hoechst 33342-positive) were the total cells. Three fields of view were randomly selected under 400-fold fields of view. Cell proliferation rate = the number of proliferating cells/the number of total cells ×100%.

### Flow Cytometry

After transfection for 48 h, the cells were detached with 0.25% trypsin. The detachment was terminated when the cells shrank and became round under microscopic observation. The cells were then gently dispersed into a cell suspension, followed by centrifugation at 1,000 rpm for 5 min with the supernatant discarded. The cells were then fixed with 70% pre-cooled ethanol for 30 min, stained with 1% propidium iodide (PI) containing RNase for 30 min, and washed twice with PBS to remove PI. The volume was adjusted to 1 mL with PBS, and the samples were placed in a BD-Aria FACS Calibur flow cytometer (FACSCalibur, Beckman Coulter Inc., 250 S. Kraemer Boulevard Brea, CA, USA) to assess cell cycle distribution.

### Monoclonal Formation Assay

Cells were seeded into the 6-well-plates and maintained in Dulbecco's modified Eagle's medium (DMEM) containing 10% FBS. Two weeks later, the cells were subject to methanol fixation and 0.1% crystal violet staining. The colonies were observed and recorded.

### Transwell Assay

Cell suspension (1 × 10^5^ cells/mL) was inoculated into the apical chamber coated with (for invasion assays) or without (for migration assays) serum-free medium-treated Matrigel and incubated in serum-free medium, while the basolateral chamber was added with medium containing 10% FBS. Twenty-four hours later, the cells in the basolateral chamber were subject to 0.1% crystal violet staining for 30 min.

### Fluorescence *In situ* Hybridization (FISH) Assay

According to the manual (Shanghai GenePharma Co., Ltd., Shanghai, China), FISH was performed using specific probes of hsa_circRPPH1_015 (5′-GTTCCAAGCTCCGGCAAA-3′) and miR-326 (5′-CTGGAGGAAGGGCCCAGAGG-3′). The cy5-labeled probe was specific for circ-ITCH while the fam-labeled probe was specific for miRNA. The nuclei were stained by 4, 6-diamino-2-phenyl indole. The slides were rinsed with PBS, fixed with 4% paraformaldehyde at room temperature, treated with protease K (RNA enzyme treatment was carried out in the control experiment to verify the specificity of the signal), and hybridized in pre-hybridizing solution with probe at 42°C overnight. After PBS containing 0.1% Tween-20 (PBST) washing, the nucleus was stained with 4′,6-diamidino-2-phenylindole (DAPI) staining solution (1:800, Thermo Fisher Scientific, Rockford, IL, USA) for 5 min, followed by three washes using PBST (3 min/time). The slides were sealed with anti-fade mounting medium, then photographed and observed in five randomly selected visual fields under a fluorescence microscope (Olympus Optical Co., Ltd., Tokyo, Japan). All images were acquired on a Zeiss LSM880 NLO (2 + 1 with BIG) confocal microscope system (Leica Microsystems, Wetzlar, Germany) (18).

### RNA Pull-Down Assay

The cells were treated with 50 nM biotin-labeled wild type (WT)-bio-miR-326 (5′-CCUCUGGGCCCUUCCTCCAG-3′) and mutation (MUT)-bio-miR-326 (5′-CCUCUGGGCCCUUTTCTTGA-3′). After 48 h of transfection, the cells were incubated in specific lysis buffer [radioimmunoprecipitation assay (RIPA) + phenylmethylsulphonyl fluoride (PMSF) + phosphatase inhibitor = 100:1:1; Ambion Company, Austin, TX, USA] for 10 min. M-280 streptavidin magnetic beads (S3762, Sigma-Aldrich, St. Louis, MO, USA) were pre-coated with RNase-free bovine serum albumin and yeast tRNA (TRNABAK-RO, Sigma-Aldrich, St. Louis, MO, USA) and used to treat cell lysate. The bound RNA was purified by Trizol and then qPCR was performed to examine the expression of hsa_circRPPH1_015.

### RNA Binding Protein Immunoprecipitation (RIP) Assay

The binding of circ_hsa_circRPPH1_015, miR-326, and Argonaute2 (Ago2) was analyzed using the RIP kit (Millipore, Billerica, MA, USA). In short, the cells were washed using pre-cooled PBS and the supernatant was discarded. The cells were then lysed using lysis buffer (25 mM Tris-HCl pH 7.4, 150 mM NaCl, 0.5% Nonidet P [NP]-40, 2 mM ethylenediaminetetraacetic acid [EDTA], 1 mM NaF, 0.5 mM dithiothreitol) comprised of RNasin (Takara Biotechnology Ltd., Dalian, Liaoning, China) and protease inhibitor mixture (B14001a, Roche, Basel, Switzerland), followed by centrifugation at 12,000 g for 30 min, with the supernatant obtained. Next, a portion of cell extract was taken out as input and a portion was incubated with antibody for co-precipitation. In brief, 50 μL magnetic beads of each co-precipitation reaction system was extracted, washed, and then re-suspended in 100 μL RIP Wash Buffer, followed by incubation with the addition of 5 μg antibodies according to the grouping for binding. Subsequently, the complex of magnetic beads-antibody was re-suspended in 900 μL RIP Wash Buffer after washing and then incubated overnight with 100 μL cell extract at 4°C. Thereafter, the samples were placed on a magnetic pedestal to collect bead-protein complex. The samples and input were detached by protease K, after which the RNA was extracted and used for subsequent RT-qPCR detection. The antibodies used in the experiment included Ago2 (ab32381, 1:50, Abcam Inc., Cambridge, UK) which was mixed evenly at room temperature for 30 min. IgG (ab109489, 1:100, Abcam Inc., Cambridge, UK) was taken as NC. RNA was then extracted from magnetic beads using Trizol and the expression of circ_hsa_circRPPH1_015 and miR-326 was assessed using RT-qPCR.

### Dual-luciferase Reporter Gene Assay

The binding among circ_hsa_circRPPH1_015, ELK1, and miR-326 was predicted using bioinformatics analysis. Dual-luciferase reporter gene assay was used to verify the predicted relationship. The full length of 3′-untranslated region (3′UTR) of circ_hsa_circRPPH1_015 and ELK1 was amplified. Then the PCR products were cloned into the polyclonal site of the reporter gene vector (Figure S1B, psiCHECK-2 purchased from Shanghai Yingbio Technology, Co., Ltd., Shanghai, China) using the endonuclease sites Spe I and Hind III. Complementary sequence mutation sites of seed sequences were designed on the circ_hsa_circRPPH1_015-WT and ELK1-WT, respectively. Next, the target fragment was inserted into the psiCHECK-2 reporter plasmid using T4 DNA ligase following restriction endonuclease. Next, the sequenced correctly luciferase reporter plasmids WT and MUT were co-transfected with mimic NC and miR-326 mimic into MCF-7 and MDA-MB-435 cells. After 24 h of transfection, the cells were collected and lysed, followed by centrifugation at 12,000 rpm for 1 min and the collection of supernatant. The luciferase activity was determined using Dual-Luciferase® Reporter Assay System (E1910, Promega, USA). The relative luciferase (RLU) activity was calculated as the RLU activity of renilla luciferase/RLU activity of firefly luciferase. The experiment was repeated three times independently (19).

### Tumor Formation in Nude Mice

Ten female nude mice (specific pathogen-free grade, Beijing HFK Bioscience Co., Ltd., Beijing, China) aged 5 weeks with weight of 18–23 g were reared at constant temperature (25–27°C) under constant humidity (45–50%). Nude mice were randomly divided into two groups (si-NC and si-circ_hsa_circRPPH1_015), five mice per group. The mice were inoculated with stably transfected cell lines after anesthesia. Cell suspension with 2 × 10^6^ cells/mL was prepared. Each nude mouse was subcutaneously injected with 0.2 mL of the single-cell suspension. On the 6th, 12th, 15th, 18th, 21st, and 24th day after inoculation, the tumor size of nude mice was measured using vernier caliper, and the tumor volume *V* (mm^3^) was estimated using the formula *V* = (*L* × *W*^2^)/2. *L* indicates the length of the tumor and *W* indicates the width of tumor. After 28 days, the nude mice were euthanized and the tumors of the nude mice were weighed. Hematoxylin-eosin (HE) staining was used to detect lung metastasis of mice.

### Immunohistochemistry

Tissue sections (5 μm) derived from xenografted tumors in nude mice were immunostained with CD34 (ab81289, Abcam Inc.), ki67 (ab16667, Abcam Inc.), and ELK1 (ab32106, Abcam Inc.). The mean intensities for positive expression of each protein product were determined by using the software Image-ProPlus 6.3 (Media Cybernetics, USA). Brown stains were defined as positive staining. The mean positive expression rate was obtained from five randomly selected fields, with a total of 200 cells in each field.

### Statistical Analysis

Obtained data were expressed as mean ± standard deviation. Comparison between two groups with normal distribution and homogeneity of variance was analyzed using the unpaired *t*-test. Data within one group were analyzed using the paired *t*-test. Comparisons among multiple groups were analyzed by one-way analysis of variance (ANOVA), followed by Tukey's *post-hoc* test. Data comparison among groups at different time points was performed by repeated-measures ANOVA, followed by Bonferroni *post-hoc* test. A value of *p* < 0.05 was regarded as statistically significant. All data were analyzed using SPSS 21.0 software (IBM, Armonk, NY, USA).

## Results

### Potential Regulatory Role of hsa_circRPPH1_015/miR-326/ELK1 Axis in BC

**Figure 1:**
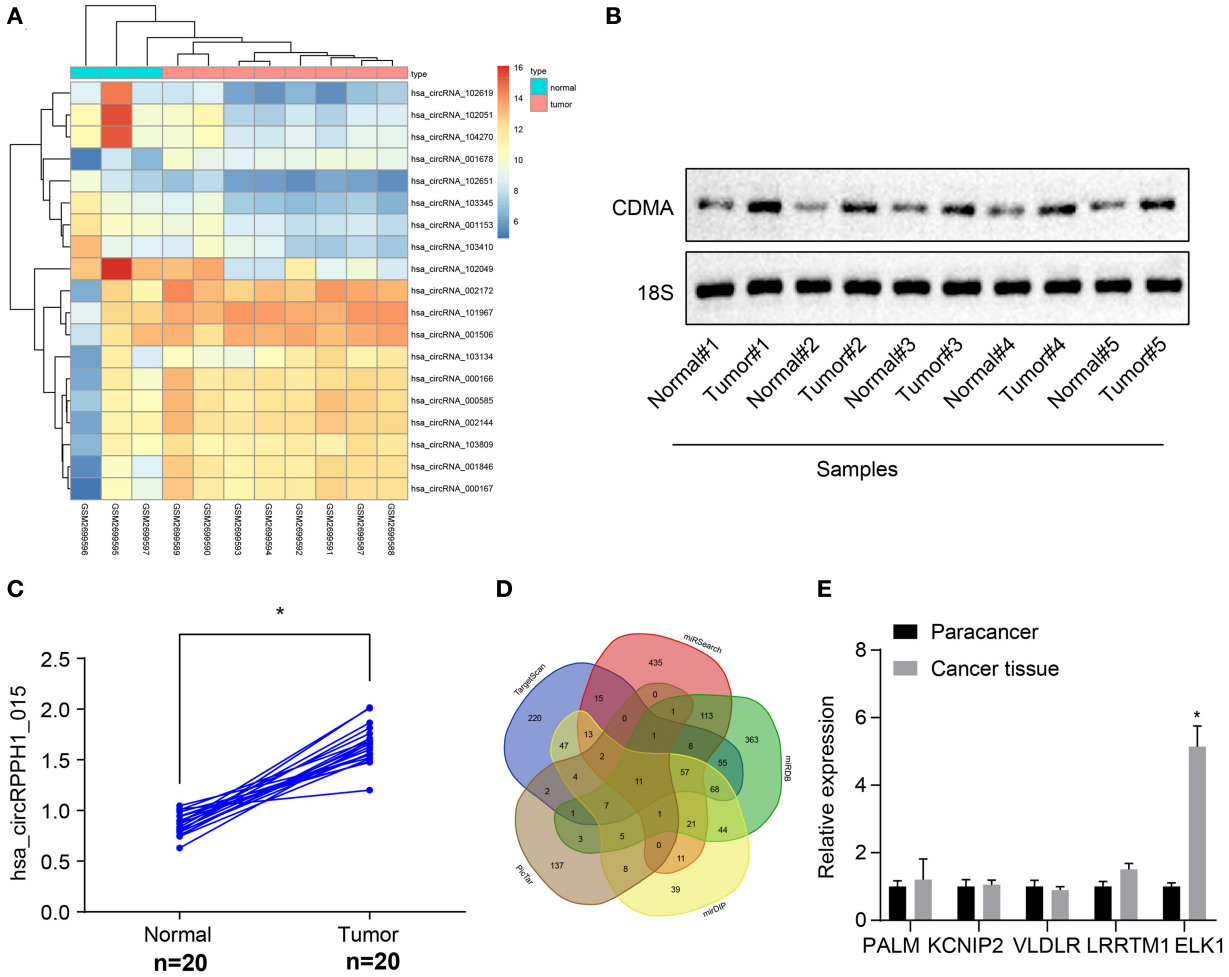
Bioinformatics revealed a possible regulatory mechanism involving hsa_circRPPH1_015, miR-326, and ELK1 in BC. **(A)** The heat map of the microarray GSE110123. **(B)** Representative image of the northern blot analyses of hsa_circRPPH1_015 expression performed using five tumor tissues and adjacent normal tissues (*N* = 5). **(C)** Quantification of northern blot analyses of hsa_circRPPH1_015 expression performed in 20 tumor tissues and adjacent normal tissues (*N* = 20). **(D)** Prediction of the target genes of miR-326 using MirDIP database, TargetScan database, miRSearch database, miRDB database, and PicTar database. **(E)** The expression of PALM, KCNIP2, VLDLR, LRRTM1, and ELK1 obtained from the GSE35412 dataset determined by RT-qPCR. *p < 0.05 vs. normal or adjacent normal tissues. The above data were all measurement data and expressed as mean ± standard deviation, and the paired *t*-test was used for comparison between cancer tissues and adjacent normal tissues. Values were obtained from three independent experiments. BC, breast cancer; ELK1, ETS-domain containing protein; KCNIP2, Kv (potassium) channel interacting protein 2; VLDLR, very low-density lipoprotein receptor; LRRTM1, leucine rich repeat transmembrane neuronal 1; RT-qPCR, reverse transcription quantitative polymerase chain reaction.

The circRNA expression data microarray GSE101123 of BC was downloaded from the Gene Expression Omnibus (GEO) database (https://www.ncbi.nlm.nih.gov/geo/). Differential analysis of the microarray data yielded 19 differentially expressed genes of BC according to the threshold of |log2FoldChange| > 2 and *p* < 0.05, of which 11 were differentially overexpressed genes and 8 were poorly expressed genes. A heat map was plotted based on the differentially expressed genes obtained from the analysis (Figure 1A). The differentially expressed circRNA hsa_circRNA_001678 with the smallest *p*-value was selected for further study, and the results demonstrated that circRNA hsa_circRNA_001678 was significantly overexpressed (Figure S2A). Besides, circBase database (http://www.circbase.org/) revealed that circRNA hsa_circRNA_001678 was also known as hsa_circ_0000517 (hsa_circRPPH1_015). Northern blot was used to detect the expression of hsa_circRPPH1_015 in breast cancer tissues and adjacent normal tissues. The results showed that the expression of hsa_circRPPH1_015 in breast cancer tissues was significantly higher than that in adjacent normal tissues (Figures 1B,C).

The targeted regulatory miRNAs of circRNA hsa_circ_0000517 were obtained from the CircInteractome database (https://circinteractome.nia.nih.gov/index.html). The miRNA with the highest context + score percentile was selected, thereby miR-326 was obtained (Figure S2C). In order to further explore the expression of miR-326 in BC, the miRNA expression data microarray GSE35412 of BC was downloaded using the GEO database. The differential analysis of the microarray showed that the differential expression of miR-326 was significant and poorly expressed in BC (Figure S2B). The target genes of miR-326 were predicted via the mirDIP database (http://ophid.utoronto.ca/mirDIP/), TargetScan database (http://www.targetscan.org/vert_71/), miRSearch database (https://www.exiqon.com/miRSearch), miRDB database (http://www.mirdb.org/), and the PicTar database (https://pictar.mdc-berlin.de/). The predicted results revealed that the intersection consisted of 11 target genes (Figure 1D; Supplementary Table 1). By comparing the predicted scores of these 11 target genes in miRDB, the first 5 genes (PALM, KCNIP2, VLDLR, LRRTM1, and ELK1) were selected for experimental verification and the differential expression of ELK1 was found to be significant (Figure 1E). The binding site between miR-326 and ELK1 was obtained through the TargetScan database (Figure S2E). The differential expression of ELK1 in BC tissues and adjacent normal tissues were examined by the GEPIA database (http://gepia.cancer-pku.cn/), and results showed that ELK1 was significantly overexpressed in BC (Figure S2D). The above results suggested that hsa_circRPPH1_015 (hsa_circ_0000517) might regulate the expression of downstream target gene ELK1 by binding to miR-326 in BC.

### Hsa_circRPPH1_015 Was Overexpressed in BC Tissues

**Figure 2:**
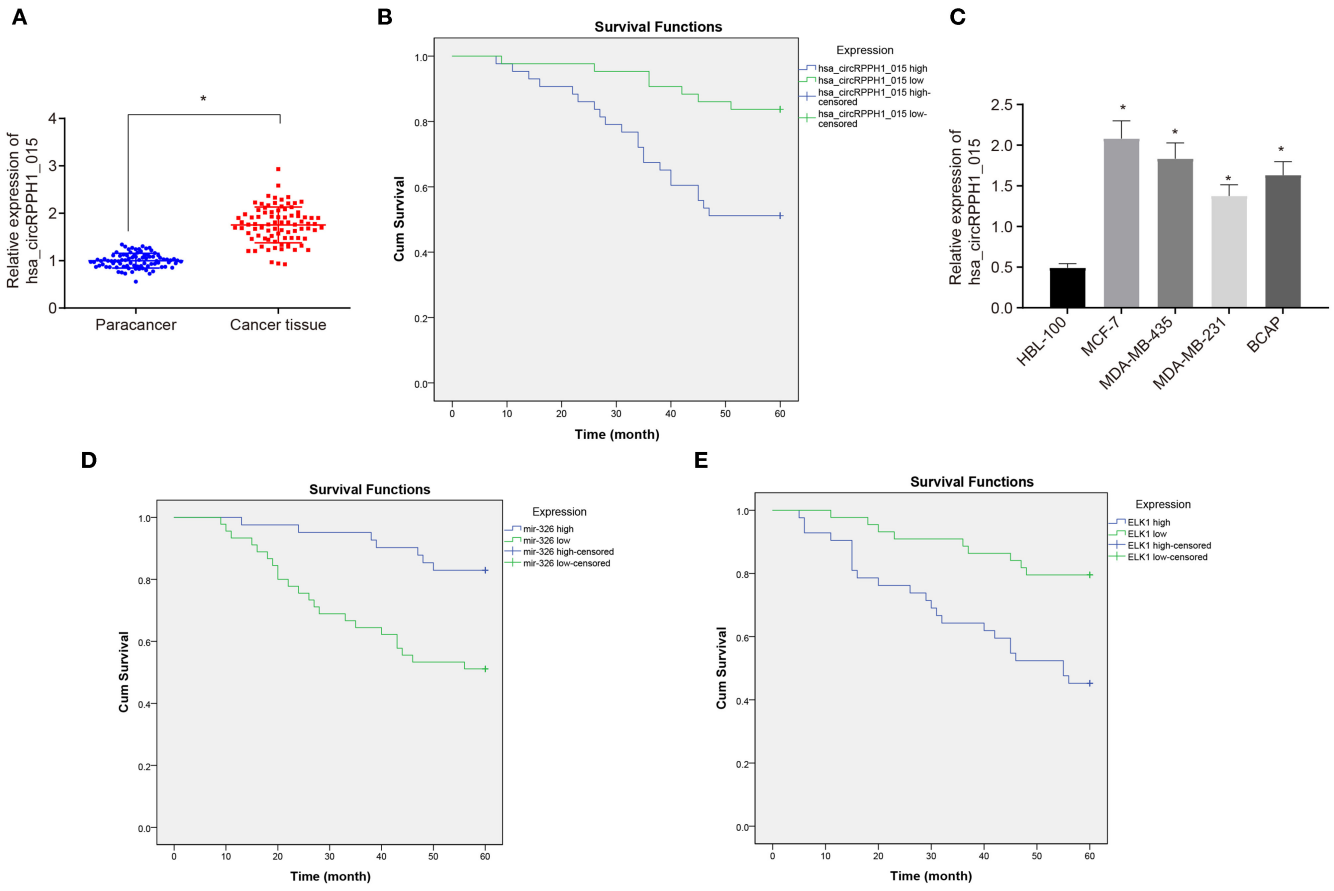
The expression of hsa_circRPPH1_015 was associated with the progression of BC. **(A)** Hsa_circRPPH1_015 expression in BC tissues and adjacent normal tissues determined by RT-qPCR. **(B)** The correlation analysis between hsa_circRPPH1_015 expression and prognosis of patients with BC by Kaplan–Meier survival analysis. **(C)** The expression of hsa_circRPPH1_015 in normal breast cell lines and BC cell lines determined by TaqMan-PCR assay. **(D)** Corelation analysis of prognosis and survival in patients with high and low expression of miR-326 by Kaplan–Meier survival analysis. **(E)** Corelation analysis of prognosis and survival in patients with high and low expression of ELK1 by Kaplan–Meier survival analysis. *p < 0.05 vs. the adjacent normal tissues or HBL-100 cell line. The quantitative analysis results were measurement data and compared by paired *t*-test between two groups and by one-way ANOVA among multiple groups. Values were obtained from three independent experiments. BC, breast cancer; ELK1, ETS-domain containing protein; RT-qPCR, reverse transcription quantitative polymerase chain reaction; ANOVA, analysis of variance.

To investigate the changes of hsa_circRPPH1_015 expression in BC tissues, RT-qPCR was used to examine the expression of hsa_circRPPH1_015 in 86 paired cancer tissues and adjacent normal tissues of patients with BC (Figure 2A). The results showed that the expression of hsa_circRPPH1_015 was significantly increased in cancer tissues when compared to the adjacent normal tissues (*p* < 0.05). The expression of hsa_circRPPH1_015 in tissues of patients with BC was analyzed, and the median value of hsa_circRPPH1_015 expression of all patients with BC was used as the cut-off value, which was further applied to analyze the relationship between hsa_circRPPH1_015 expression and clinicopathological features of patients with BC. The results (Table 2) showed that hsa_circRPPH1_015 expression was significantly elevated in BC patients with increased tumor diameters, higher stage, and grade and lymph node metastasis. Postoperative follow-up of BC patients revealed that patients with low expression of hsa_circRPPH1_015 have survived for a longer period (Figure 2B). Moreover, the expression of hsa_circRPPH1_015 was significantly increased in BC cell lines in comparison to normal breast cell line HBL-100 (*p* < 0.05), where the expression of hsa_circRPPH1_015 was the highest in MCF-7 cell lines (Figure 2C). At the same time, we also analyzed the relationship between the expression of miR-326 and ELK1 and the survival rate of BC patients, and found that the patients with high expression of miR-326 (Figure 2D) and low expression of ELK1 (Figure 2E) had better prognosis.

**Table 2:** Relationship between hsa_circRPPH1_015 and clinicopathological features of patients.

**Clinicopathological features**	**Case**	**Low**	**High**	***p*-value**
**Age**				0.268
<50	33	19 (57.58%)	14 (42.42%)	
≥50	53	24 (45.28%)	29 (54.72%)	
**Menopausal state**				0.372
Premenopausal	54	25 (46.29%)	29 (53.72%)	
Late menopause	32	18 (56.25%)	14 (43.75%)	
**Tumor size (cm)**				0.024
<2	40	19 (47.50%)	21 (52.50%)	
2–5	30	20 (66.67%)	10 (33.33%)	
>5	16	4 (25.00%)	12 (75.00%)	
**Clinical stage**				<0.001
I	44	19 (43.18%)	25 (56.82%)	
II	27	22 (81.48%)	5 (18.52%)	
IIIa	15	2 (13.33%)	13 (86.67%)	
**Pathological grading**				0.029
I	40	20 (50.00%)	20 (50.00%)	
II	25	17 (68.00%)	8 (32.00%)	
III	21	6 (28.57%)	15 (71.43%)	
**Lymph node metastasis**				0.001
Yes	51	18 (35.29%)	33 (64.71%)	
No	35	25 (71.43%)	10 (28.57%)	

*The data are counting data, and expressed by n (%) and chi-square test was carried out*.

### Hsa_circRPPH1_015 Promotes the Development of BC

In order to observe the effect of hsa_circRPPH1_015 on the development of BC, relevant experiments were performed on the MCF-7 cells. Since hsa_circRPPH1_015 was highly expressed in BC tissues and cell lines, the effect of hsa_circRPPH1_015 on the biological characteristics of BC was further investigated by down-regulating the expression of hsa_circRPPH1_015 in BC cell line MCF-7. The relative expression of hsa_circRPPH1_015 and linear hsa_RPPH1_015 was examined by RT-qPCR using siRNA that silenced hsa_circRPPH1_015.

**Figure 3:**
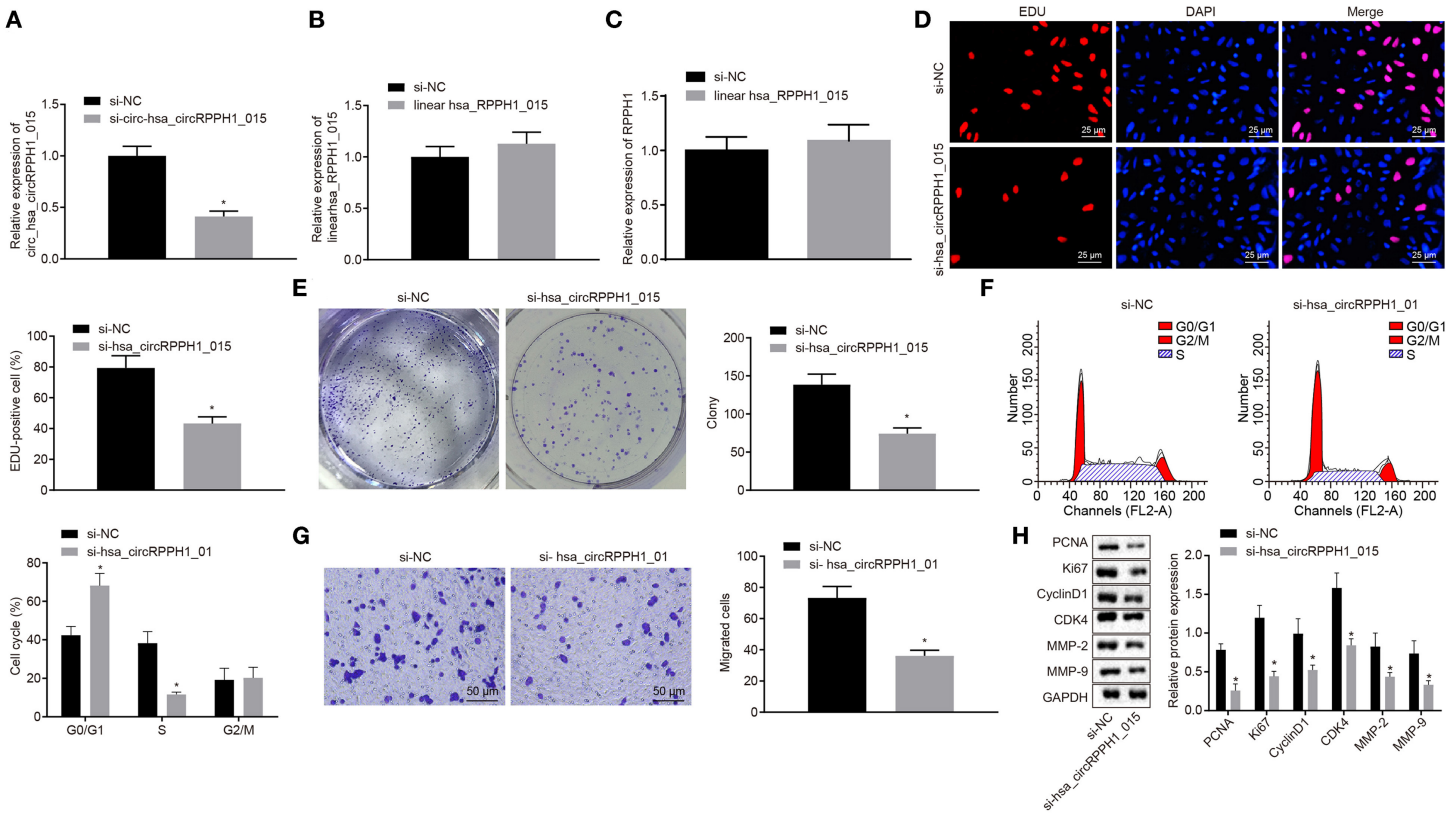
Silencing of hsa_circRPPH1_015 exerts inhibitory effects on the proliferation and invasion of BC cells. **(A)** The relative expression of hsa_circRPPH1_015 in MCF-7 cells examined by TaqMan-PCR assay. **(B)** The relative expression of linear hsa_RPPH1_015 in MCF-7 cells examined by RT-qPCR. **(C)** The relative expression of ELK1 in MCF-7 cells examined by RT-qPCR. **(D)** MCF-7 cell proliferation assessed by EdU. **(E)** Representative imges of MCF-7 colony formation and the quantification diagram examined by colony formation assay. **(F)** MCF-7 cell cycle examined by flow cytometry. **(G)** MCF-7 cell invasion examined by Transwell assay. **(H)** The expression of related proteins in MCF-7 cells normalized to GAPDH assessed by Western blot analysis. *p < 0.05 vs. the si-NC group (MCF-7 cells transfected with si-NC). The quantitative analysis results were measurement data and compared by unpaired *t*-test between two groups and by one-way ANOVA among multiple groups. Values were obtained from three independent experiments. BC, breast cancer; ELK1, ETS-domain containing protein; EdU, 5-ethynyl-2′-deoxyuridine; RT-qPCR, reverse transcription quantitative polymerase chain reaction; GAPDH, glyceraldehyde-3-phosphate dehydrogenase; si-NC, small interfering RNA-negative control; ANOVA, analysis of variance.

RT-qPCR results showed that the relative expression of circ-hsa_circRPPH1_015 was significantly decreased by si-circ-hsa_circRPPH1_015, while no significant difference was observed in the relative expression of linear hsa_RPPH1_015 (Figures 3A,B), indicating that the regulatory role in this study may be induced by hsa_circRPPH1_015 instead of linear hsa_RPPH1_015. Since circRNA may affect the transcription of the parent gene, we detected the expression of RPPH1 after interfering hsa_circRPPH1_015 expression, and the results showed that there was no significant difference in the expression of RPPH1 after interfering hsa_circRPPH1_015 expression (Figure 3C). The EdU assay (Figure 3D) and the colony formation experiment (Figure 3E) demonstrated that the proliferation ability of the MCF-7 cells transfected with si-hsa_circRPPH1_015 was significantly decreased as compared to the MCF-7 cells transfected with si-NC, while the volume and size of the clone formation also significantly decreased (*p* < 0.05). The results of the cell cycle progression detected by flow cytometry (Figure 3F) showed that the number of cells arrested in G0/G1 phase in response to si-hsa_circRPPH1_015 was significantly higher, and the number of cells arrested in S phase was significantly lower (*p* < 0.05). Furthermore, Transwell results (Figure 3G) revealed that the invasive ability of the MCF-7 cells transfected with si-hsa_circRPPH1_015 was significantly suppressed (*p* < 0.05). Western blot analysis of the expression of related protein factors (Figure 3H) demonstrated that the expression of PCNA, Ki67, Cyclin D1, MMP-2, MMP-9, and CDK4 in MCF-7 cells transfected with si-hsa_circRPPH1_015 was significantly lower (*p* < 0.05). The above results indicated that silencing of hsa_circRPPH1_015 could inhibit the proliferation and invasion of BC cells.

### Hsa_circRPPH1_015 Regulated miR-326

**Figure 4:**
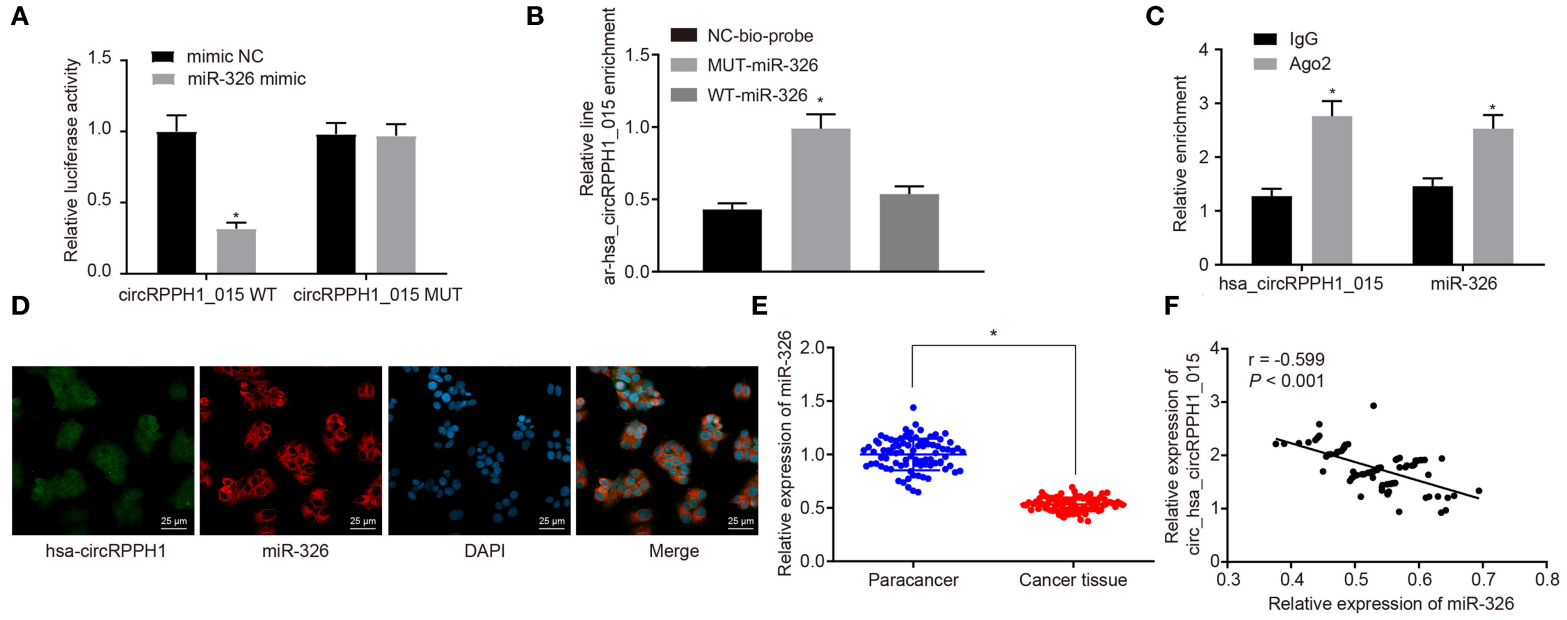
The miR-326 expression was inhibited by hsa_circRPPH1_015. **(A)** Verification of the binding between hsa_circRPPH1_015 and miR-326 by dual-luciferase reporter gene assay in MCF-7 cells, *p < 0.05 vs. the si-NC group (MCF-7 cells transfected with si-NC). **(B)** The linear-hsa_circRPPH1_015 enrichment relative to NC-bio-probe detected by RNA pull-down experiment in MCF-7 cells, *p < 0.05 vs. the NC-bio-probe group (MCF-7 cells treated with NC-bio-probe). **(C)** The enrichment of Ago2 relative to IgG detected by RIP experiment in MCF-7 cells, *p < 0.05 vs. the IgG group (MCF-7 cells treated with IgG). **(D)** The cellular localization of hsa_circRPPH1_015 and miR-326 tested by FISH in MCF-7 cells. **(E)** The expression of miR-326 in adjacent normal tissues and BC tissues assessed by RT-qPCR, *p < 0.05 vs. the adjacent normal tissues. **(F)** Pearsons' analysis of the correlation between hsa_circRPPH1_015 and miR-326. The quantitative analysis results were measurement data and compared by paired or unpaired *t*-test between two groups and by one-way ANOVA among multiple groups. Values were obtained from three independent experiments. BC, breast cancer; ELK1, ETS-domain containing protein; RT-qPCR, reverse transcription quantitative polymerase chain reaction; IgG, immunoglobulin G; RIP, RNA binding protein immunoprecipitation; si-NC, small interfering RNA-negative control; ANOVA, analysis of variance.

To investigate the relationship between hsa_circRPPH1_015 and miR-326, the binding site between hsa_circRPPH1_015 to miR-326 was predicted using the website circinteractome.nia.nih.gov (Figure S3A). The results of the dual-luciferase reporter gene assay (Figure 4A; Figure S3B) showed that the luciferase activity of miR-326-WT was inhibited by silencing of hsa_circRPPH1_015 (*p* < 0.05), whereas the luciferase activity of miR-326-MUT was not affected (*p* > 0.05), indicating that hsa_circRPPH1_015 could specifically bind to miR-326. The RNA pull-down assay (Figure 4B; Figure S3C) demonstrated a significant increase in hsa_circRPPH1_015 binding to WT-miR-326 compared with binding to MUT-miR-326 (*p* < 0.05), suggesting that hsa_circRPPH1_015 could be directly combine with miR-326. The RIP experiment revealed that Ago2-bound hsa_circRPPH1_015 was significantly increased (*p* < 0.05) compared to IgG, suggesting that hsa_circRPPH1_015 could bind to Ago2 protein (Figure 4C; Figure S3D). To further explore the interaction between hsa_circRPPH1_015 and miR-326, the co-localization of hsa_circRPPH1_015 and miR-326 in cells was explored by FISH assay. Results (Figure 4D; Figure S3E) showed that hsa_circRPPH1_015 was co-localized with miR-326 in BC cells.

RT-qPCR (Figure 4E) was used to examine the expression of miR-326 in adjacent normal tissues and BC tissues. The results showed that the expression of miR-326 was lower in BC tissues than in adjacent normal tissues (*p* < 0.05). The correlation analysis between hsa_circRPPH1_015 and miR-326 revealed that hsa_circRPPH1_015 was negatively correlated with miR-326 (Figure 4F). The aforementioned findings suggested that hsa_circRPPH1_015 exert a regulatory role in the expression of miR-326.

### miR-326 Inhibited the Development of BC

**Figure 5:**
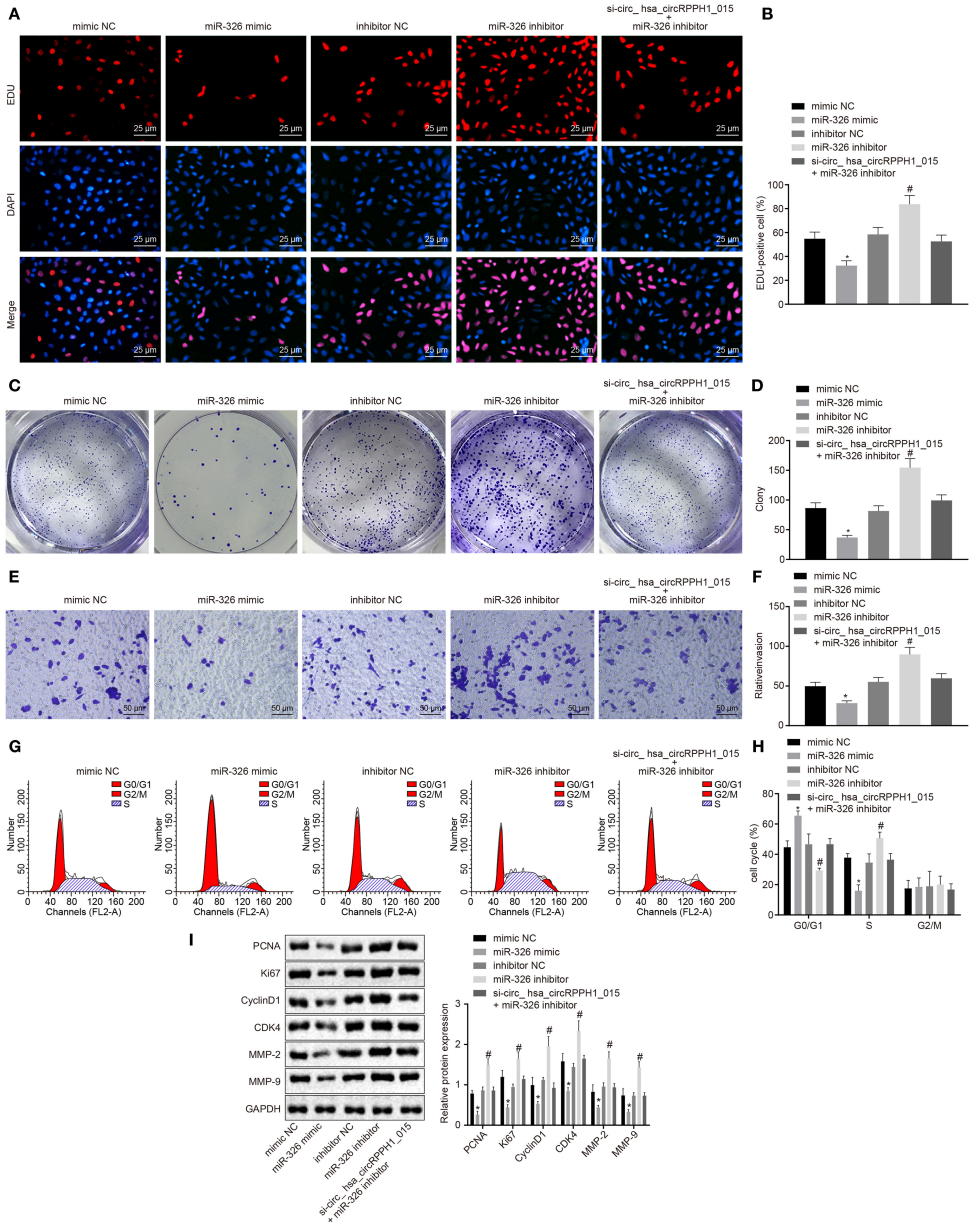
miR-326 inhibited the occurrence and development of BC. **(A,B)** MCF-7 cells positive staining in each group examined by EdU assay (×400). **(C,D)** Representative images of MCF-7 colony formation and the quantification diagram examined by colony formation assay. **(E,F)** The invasion ability of MCF-7 cells in each group examined by Transwell assay (×200). **(G,H)** The cell cycle distribution of MCF-7 cells in each group examined by flow cytometry. **(I)** The relative protein expression of associated proteins normalized to GAPDH in each group determined by Western blot analysis in MCF-7 cells. *p < 0.05 vs. the mimic-NC group (MCF-7 cells transfected with mimic-NC). #*p* < 0.05 vs. the inhibitor-NC group (MCF-7 cells transfected with inhibitor-NC). The quantitative analysis results were measurement data and analyzed by one-way ANOVA among multiple groups. Values were obtained from three independent experiments. BC, breast cancer; EdU, 5-ethynyl-2′-deoxyuridine; GAPDH, glyceraldehyde-3-phosphate dehydrogenase; NC, negative control; ANOVA, analysis of variance.

To further investigate whether the alternation of miR-326 affects the development and progression of BC, the EdU (Figures 5A,B; Figures S4A,B), plate colony formation experiment (Figures 5C,D; Figures S4C,D), Transwell (Figures 5E,F; Figures S4E,F), and flow cytometry assay (Figures 5G,H; Figures S4G,H) were conducted to examine the proliferation, colony-forming ability, invasion ability, and cell cycle distribution. Western blot analysis was performed to examine the expression of related proteins for further verification (Figure 5I; Figure S4I). The results showed that compared with the MCF-7 cells transfected with mimic-NC, MCF-7 cells transfected with miR-326 mimic exhibited significant suppression in cell proliferation, colony formation, and invasion ability, more G0/G1 phase-arrested cells, and fewer S phase-arrested cells, but decrease in expression of PCNA, Ki67, Cyclin D1, MMP-2, MMP-9, and CDK4 (*p* < 0.05). However, the transfection of miR-326 inhibitor induced opposite changing tendency (*p* < 0.05). These results indicated that miR-326 overexpression could suppress the proliferation, invasion, and angiogenesis of BC cells, as well as eliminate the pro-cancer effect caused by hsa_circRPPH1_015.

### Hsa_circRPPH1_015 Regulates ELK1 Expression by Binding to miR-326

**Figure 6:**
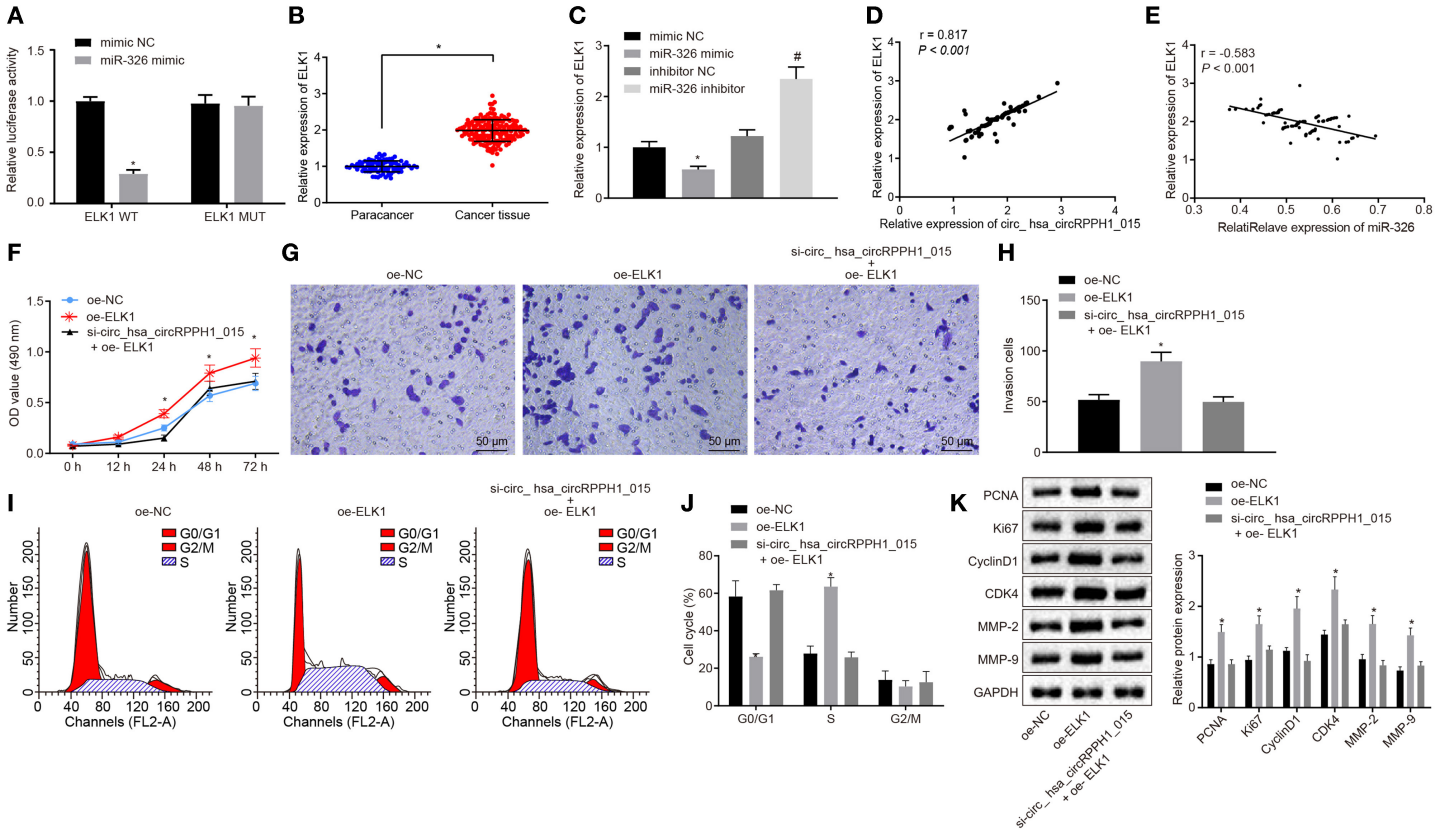
Regulation of ELK1 via miR-326 by hsa_circRPPH1_015 contributes to the development of BC. **(A)** The targeting relationship between miR-326 and ELK1 verified by dual-luciferase reporter gene assay. **(B)** The expression of ELK1 in BC tissues (*n* = 86) and adjacent normal tissues (*n* = 86) examined by RT-qPCR. **(C)** The expression of ELK1 in MCF-7 cells of each group examined by RT-qPCR. **(D)** The correlation analysis between hsa_circRPPH1_015 and ELK1. **(E)** The correlation analysis between miR-326 and ELK1. **(F)** The proliferation of MCF-7 cells in each group assessed by EdU assay. **(G,H)** The cell migration ability of MCF-7 assessed by Transwell assay (×200). **(I,J)** The distribution of MCF-7 cell cycle in each group detected by flow cytometry. **(K)** The expression of associated proteins normalized to GAPDH in each group determined by Western blot analysis. *p < 0.05 vs. the mimic NC group (MCF-7 cells transfected with mimic NC). #*p* < 0.05 vs. the si-NC group (MCF-7 cells transfected with si-NC), the oe-NC group (MCF-7 cells transfected with oe-NC), or the inhibitor NC group (MCF-7 cells transfected with inhibitor NC). The quantitative analysis results were measurement data and compared by unpaired *t*-test between two groups and by one-way ANOVA among multiple groups. Values were obtained from three independent experiments. BC, breast cancer; ELK1, ETS-domain containing protein; EdU, 5-ethynyl-2′-deoxyuridine; RT-qPCR, reverse transcription quantitative polymerase chain reaction; GAPDH, glyceraldehyde-3-phosphate dehydrogenase; si-NC, small interfering RNA-negative control; oe, overexpression; ANOVA, analysis of variance.

Bioinformatics analysis revealed the presence of binding site between miR-326 and ELK1 (Figure S5A). Results from dual-luciferase reporter gene assay verified that co-transfection of miR-326 mimic with ELK1-WT 3′-UTR resulted in strongly reduced luciferase activity, whereas there was no significant difference witnessed after co-transfection with ELK1-MUT 3′-UTR (Figure 6A; Figure S5A). The expression of ELK1 in BC tissues and adjacent normal tissues was examined by RT-qPCR. Results (Figure 6B) demonstrated that the expression of ELK1 in BC tissues was significantly higher than that in adjacent normal tissues. The expression of ELK1 in MCF-7 cells transfected with mimic-NC, miR-326 mimic, inhibitor-NC, and miR-326 inhibitor was examined by RT-qPCR (Figure 6C; Figure S5B). The results revealed that the expression of ELK1 was significantly reduced in MCF-7 cells transfected with miR-326 mimic, while significantly elevated in MCF-7 cells transfected with miR-326 inhibitor.

The correlation analysis showed a positive correlation between hsa_circRPPH1_015 and ELK1 (Figure 6D; Figure S5C), whereas a negative correlation between miR-326 and ELK1 was observed (Figure 6E; Figure S5D). In order to test whether hsa_circRPPH1_015 exerts cancer-promoting effect via regulation of ELK1, a target gene of miR-326, cell proliferation (Figure 6F; Figure S5E), invasion (Figures 6G,H; Figures S5F,G), and cell cycle distribution (Figures 6I,J; Figures S5H,I) were examined by EdU, Transwell, and flow cytometry, respectively. Western blot analysis was performed to assess the expression of related proteins (Figure 6K; Figure S5J). The results showed that the proliferation and invasion of MCF-7 cells transfected with oe-ELK1 were significantly increased, with fewer cells arrested in G0/G1 phase and more cells arrested in S phase (*p* < 0.05). The expression of PCNA, Ki67, MMP-2, MMP-9, Cyclin D1, and CDK4 were increased in each group. Besides, even when hsa_circRPPH1_015 was silenced, the overexpression of ELK1 was sufficient to promote the proliferation and invasion and induce cell cycle arrest of MCF-7 cells in S phase. The above results suggested that hsa_circRPPH1_015 could exert cancer-promoting effect by regulating ELK1 via miR-326.

### Down-Regulation of hsa_circRPPH1_015 Inhibits Xenograft Tumorigenesis in Nude Mice

**Figure 7:**
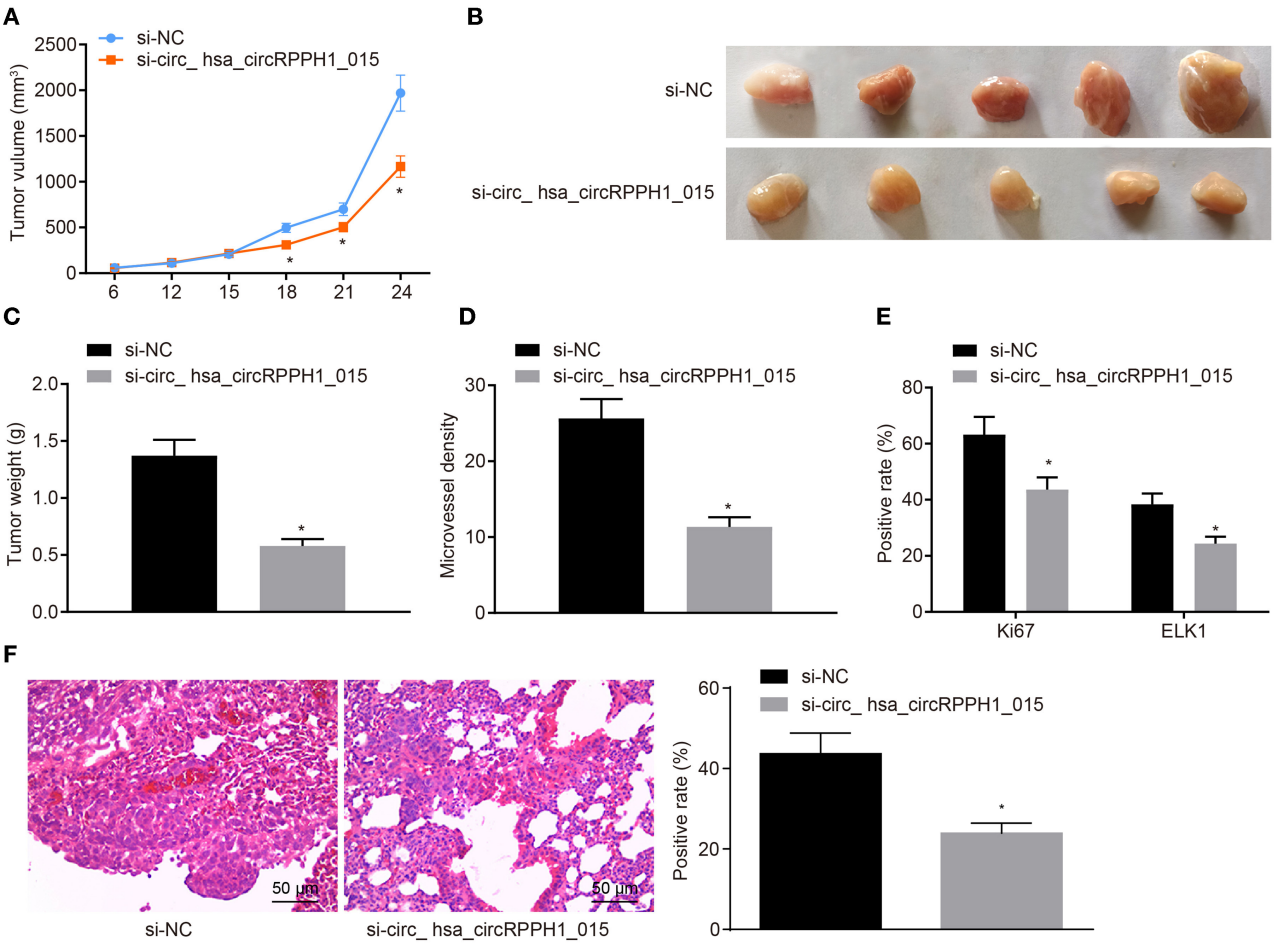
Silencing of hsa_circRPPH1_015 inhibits xenograft tumorigenesis in nude mice. **(A)** Tumor volume in nude mice. **(B)** Representative image of xenograft tumor formation in nude mice. **(C)** Tumor weights in nude mice. **(D)** MVD of nude mice in each group identified by immunohistochemistry. **(E)** The positive rates of Ki67 and ELK1 in tumors of nude mice tested by Immunohistochemistry. **(F)** The number of lung metastases of tumor cells after interferencing hsa_circRPPH1_015 expression by HE staining. *p < 0.05 vs. the si-NC group (nude mice bearing MCF-7 cells transfected with si-NC), *n* = 10. The quantitative analysis results were measurement data and compared by unpaired *t*-test between two groups and by one-way ANOVA among multiple groups. Values were obtained from three independent experiments. MVD, micro-vessel density; ELK1, ETS-domain containing protein; HE, Hematoxylin-eosin; si-NC, small interfering RNA-negative control; ANOVA, analysis of variance.

We have previously confirmed that hsa_circRPPH1_015 participated in the development of BC by binding to miR-326 and regulating ELK1 *in vitro*. To further investigate whether the down-regulated hsa_circRPPH1_015 could inhibit tumor growth *in vivo*, MCF-7 cells transfected with si-NC and si-hsa_circRPPH1_015 were subcutaneously injected into nude mice. Tumor volume (Figure 7A) and tumor weight (Figures 7B,C) were measured after injection. Micro-vessel density (MVD) (Figure 7D) and positive rates of Ki67 and ELK1 expression were examined by immunohistochemistry (Figure 7E). The results demonstrated that the volume and weight of nude mice in the presence of si-hsa_circRPPH1_015 was significantly decreased. Besides, both Ki67 and ELK1 showed significant decreases in their positive rates (*p* < 0.05). HE staining of lung tissues showed that down regulating hsa_circRPPH1_015 could inhibit the number of lung metastases of tumor cells (Figure 7F). These findings elucidated that the tumor formation in nude mice could be inhibited by the down-regulated hsa_circRPPH1_015.

## Discussion

Breast cancer (BC) is one of the top three cancers across the world with different treatment modes depending on the different subtypes (4). However, the current applicable treatment for BC exerts a considerable large and prolonged impact on the activity levels of BC survivors (20). This study aims to explore the potential regulatory role of hsa_circRPPH1_015 in the development of BC. The present study has demonstrated that hsa_circRPPH1_015 could regulate the expression of ELK1 in BC cells by binding to miR-326 and thereby contributes to the progression of BC.

The results from the present study revealed that hsa_circRPPH1_015 was overexpressed in BC cells and the silencing of hsa_circRPPH1_015 resulted in inhibition of growth, invasion, and cell cycle progression of BC cells. A group of circRNAs has been shown to play oncogenic roles in BC progression. Consistent with our findings, the overexpression of hsa_circ_0006528 has been reported in human BC tissues and thereby promotes cell proliferation, invasion, and migration (21). CircAGFG1 promotes the proliferation, invasion, and mobility along with metastasis and tumorigenesis of triple-negative BC cells (22). The overexpression of hsa_circ_0052112 has been indicated to facilitate the cell migration and invasion of BC in previous finding (7). Besides, silencing of siRNA-mediated circ-UBE2D2 has been shown to have potency to impede the proliferation, migration, and invasion of BC cells (23). Emerging evidence has also shown that Ki67 predicts cancer progression as a marker of cell proliferation that highly expressed Ki67 has been noted in cancer cells (24). The overexpressed Cyclin D1 has been shown to play oncogenic role in endometrial carcinoma and was related to diagnosis, prognosis, and survival of patients (25). MMPs have been reported to be involved in physiological and pathological processes of many diseases (26). In the context of cancers, MMPs has been reported to be highly expressed through all stages (27). CDK4 inhibitors are a new class of anti-cancer drugs that commonly used in the treatment of metastatic BC (28). Consistently, the present study demonstrated that silencing of hsa_circRPPH1_015 could inhibit the growth and invasion of BC cells by suppressing the expression of PCNA, Ki67, Cyclin D1, MMP-2, MMP-9, and CDK4. Besides, the *in vivo* experiments have indicated that silencing of hsa_circRPPH1_015 could inhibit xenograft tumorigenesis of MCF-7 cells in nude mice.

As our experiment revealed, miR-326 was down-regulated in BC tissues or cells, while the overexpression of miR-326 resulted in inhibition of proliferation, invasion, and angiogenesis of BC cells. Likewise, previous evidence has shown that miR-326 may act as an anti-tumor miR in the occurrence and development of human prostatic carcinoma (10). Consistent with our findings, miR-326 was down-regulated in the tissues and cell lines of human BC, and the overexpressed miR-326 has been showed to inhibit the cell proliferation, migration, and invasion of BC (11). Besides, miR-326 has been reported to be poorly expressed in gastric cancer cells and overexpression of miR-326 inhibits the proliferation of gastric cancer cells (29). A functional study has proposed that circRNAs are able to bind to miRNAs (30). Similarly, hsa_circ_0003998 has been reported to promote the proliferation and invasion of non-small cell lung cancer cells by targeting miR-326 (31). Long non-coding RNA small nucleolar RNA host gene 1 has been previously reported to regulate the NOB1 expression by binding to miR-326 in osteosarcoma (32). Those findings have supported the regulatory mechanism that silencing of hsa_circRPPH1_015 could up-regulate the expression of miR-326 to inhibit the proliferation, invasion, and angiogenesis of BC cells.

In addition, our finding suggested that ELK1 was up-regulated in BC tissues or cells and may play a tumor-promoting role in BC. Consistent with our findings, the up-regulation of ELK1 has been previously reported in hepatocellular carcinoma tissues while the depletion of ELK1 exerts an inhibitory effect on cell migration and invasion (33). ELK1 has also been elucidated to play a crucial role in the tumorigenesis and progression of bladder cancer (34). Besides, the growth of BC cells has been shown to be promoted by ELK1 and ELK3, both of which were the target genes of miR-135a and critical regulators of tumor-suppressive effects induced by miR-135a (14). The present study also revealed that ELK1 could be targeted and negatively regulated by miR-326. In agreement with our experimental findings, miR-326 has been previously reported to inhibit cervical cancer cell proliferation, migration, and invasion by targeting ELK1 (35). The above-mentioned literatures suggested that miR-326 could inhibit the progression of BC by targeting ELK1.

## Conclusion

In short, our study demonstrated that the silencing of hsa_circRPPH1_015 could up-regulate the expression of miR-326 and down-regulate the expression of ELK1, thus reducing the proliferation, colony formation, and invasion ability of BC cells. Collectively, these findings suggested that hsa_circRPPH1_015 could increase the expression of ELK1 by binding to miR-326, highlighting the potential detrimental contribution of hsa_circRPPH1_015 to BC. The present study provides new insights for the clinical therapy of BC. However, more in-depth investigation underlying the potential regulatory pathway of ELK1 is required in the future.

## Data Availability Statement

The datasets analysed in this study can be found in the NCBI Gene Expression Omnibus (GSE101123, GSE35412).

## Ethics Statement

This study was approved by the Ethics Committee of Shandong Provincial Hospital Affiliated to Shandong First Medical University and conformed to the Helsinki Declaration. All patients signed informed consent forms. The animal experiments were performed with the approval of the Animal Ethics Committee.

## Author Contributions

CZ, LL, and ZL designed the study. JX, QY, and PS collated the data, carried out data analyses, and produced the initial draft of the manuscript. CZ, KZ, and RJ contributed to drafting the manuscript. All authors have read and approved the final submitted manuscript.

## Conflict of Interest

The authors declare that the research was conducted in the absence of any commercial or financial relationships that could be construed as a potential conflict of interest.

## Supplementary Material

Figure S1 Schematic diagram of primers that distinguishing circRNA RNA and linear RNA **(A)** and plasmid structure of psiCHECK-2 **(B)**.

Figure S2 Bioinformatics revealed a possible regulatory mechanism involving hsa_circRPPH1_015 in BC. **(A)** The expression of hsa_circRNA_001678 in the microarray GSE110123. **(B)** The expression of miR-326 in tumor tissues and adjacent normal tissues in BC. **(C)** Prediction of the target miRNA of hsa_circRNA_001678 (hsa_circ_0000517) using CircInteractome database. **(D)** ELK1 expression in adjacent normal and tumor tissues in TCGA database, the abscissa represents the disease name and samples, the ordinate represents the TPM value, the red box graph represents the tumor sample, and the gray box graph represents the normal sample. **(E)** Binding sites between miR-326 and ELK1 predicted by the TargetScan database. BC, breast cancer; ELK1, ETS-domain containing protein; TCGA, the Cancer Genome Atlas; TPM, transcripts per million.

Figure S3 The miR-326 expression was inhibited by hsa_circRPPH1_015. **(A)** Prediction of the binding between hsa_circRPPH1_015 and miR-326. **(B)** Verification of the binding between hsa_circRPPH1_015 and miR-326 by dual-luciferase reporter gene assay in MDA-MB-435 cells, ^*^*p* < 0.05 vs. the si-NC group (MDA-MB-435 cells transfected with si-NC). **(C)** The linear-hsa_circRPPH1_015 enrichment relative to NC-bio-probe detected by RNA pull-down experiment in MDA-MB-435 cells, ^*^*p* < 0.05 vs. the NC-bio-probe group (MDA-MB-435 cells treated with NC-bio-probe). **(D)** The enrichment of Ago2 relative to IgG detected by RIP experiment in MDA-MB-435 cells, ^*^*p* < 0.05 vs. the IgG group (MDA-MB-435 cells treated with IgG). **(E)** The cellular localization of hsa_circRPPH1_015 and miR-326 tested by FISH in MDA-MB-435 cells. The quantitative analysis results were measurement data and compared by paired or unpaired *t*-test between two groups and by one-way ANOVA among multiple groups. Values were obtained from three independent experiments. si-NC, small interfering RNA-negative control; RIP, RNA binding protein immunoprecipitation; FISH, fluorescence *in situ* hybridization; IgG, immunoglobulin G; ANOVA, analysis of variance.

Figure S4 miR-326 inhibited the occurrence and development of BC. **(A,B)** MDA-MB-435 cell positive staining in each group examined by EdU assay. **(C,D)** Representative images of MDA-MB-435 colony formation and the quantification diagram examined by colony formation assay. **(E,F)** The invasion ability of MDA-MB-435 cells in each group examined by Transwell assay. **(G,H)** The cell cycle distribution of MDA-MB-435 cells in each group examined by flow cytometry. **(I)** The relative protein expression of associated proteins normalized to GAPDH in each group determined by Western blot analysis in MDA-MB-435 cells. ^*^*p* < 0.05 vs. the mimic-NC group (MDA-MB-435 cells transfected with mimic-NC). #*p* < 0.05 vs. the inhibitor-NC group (MDA-MB-435 cells transfected with inhibitor-NC). The quantitative analysis results were measurement data and analyzed by one-way ANOVA among multiple groups. Values were obtained from three independent experiments. BC, breast cancer; EdU, 5-ethynyl-2′-deoxyuridine; GAPDH, glyceraldehyde-3-phosphate dehydrogenase; NC, negative control; ANOVA, analysis of variance.

Figure S5 Regulation of ELK1 via miR-326 by hsa_circRPPH1_015 contributes to the development of BC. **(A)** The targeting relationship between miR-326 and ELK1 verified by dual-luciferase reporter gene assay in MDA-MB-435 cells. **(B)** The expression of ELK1 in MDA-MB-435 cells examined by RT-qPCR. **(C)** The correlation analysis between hsa_circRPPH1_015 and ELK1. **(D)** The correlation analysis between miR-326 and ELK1. **(E)** The proliferation of MDA-MB-435 cells in each group assessed by EdU assay. **(F,G)** The cell migration ability of MDA-MB-435 cells assessed by Transwell assay. **(H,I)** The distribution of MDA-MB-435 cell cycle in each group detected by flow cytometry. **(J)** The expression of associated proteins normalized to GAPDH in each group determined by Western blot analysis. ^*^*p* < 0.05 vs. the mimic NC group (MDA-MB-435 cells transfected with mimic NC). #*p* < 0.05 vs. the inhibitor NC group (MDA-MB-435 cells transfected with inhibitor NC), the oe-NC group (MDA-MB-435cells transfected with oe-NC). The quantitative analysis results were measurement data and compared by unpaired *t*-test between two groups and by one-way ANOVA among multiple groups. Values were obtained from three independent experiments. BC, breast cancer; ELK1, ETS-domain containing protein; EdU, 5-ethynyl-2′-deoxyuridine; RT-qPCR, reverse transcription quantitative polymerase chain reaction; GAPDH, glyceraldehyde-3-phosphate dehydrogenase; NC, negative control; oe, overexpression; ANOVA, analysis of variance.

Supplementary Table 1 Predicted target genes of miR-326.

## Abbreviations

BC: Breast cancer
circRNAs: circular RNAs
ELK1: ETS-domain containing protein
FBS: fetal bovine serum
miRNAs: microRNAs
ELK1: ETS-domain containing protein
ATCC: American Type Culture Collection
RPMI: Roswell Park Memorial Institute
RT-qPCR: Reverse transcription quantitative polymerase chain reaction
si: small interfering RNA
oe: overexpression
NC: negative control
MEM: minimum essential medium
SDS-PAGE: sodium dodecyl sulfate polyacrylamide gel electrophoresis
cDNA: complementary DNA
EP: Eppendorf
GAPDH: glyceraldehyde-3-phosphate dehydrogenase
PCNA: proliferating cell nuclear antigen
MMP: matrix metalloproteinase
CDK4: Cyclin-dependent kinase 4
EdU: 5-ethynyl-2′-deoxyuridine
PI: propidium iodide
PBS: phosphate-buffered saline
DMEM: Dulbecco's modified Eagle's medium
FISH: Fluorescence *in situ* hybridization
PBST: PBS containing 0.1% Tween-20
DAPI: 4′,6-diamidino-2-phenylindole
WT: wild type
MUT: mutation
RIPA: radioimmunoprecipitation assay
PMSF: phenylmethylsulphonyl fluoride
RIP: RNA binding protein immunoprecipitation
Ago2: Argonaute2
NP: Nonidet P
EDTA: ethylenediaminetetraacetic acid
3′UTR: 3′-untranslated region
RLU: relative luciferase
HE: Hematoxylin-eosin
ANOVA: analysis of variance
GEO: Gene Expression Omnibus.
