# Supplementary figures and images for: A Novel Circular RNA hsa_circRPPH1_015 Exerts an Oncogenic Role in Breast Cancer by Impairing miRNA-326-Mediated ELK1 Inhibition
Source: Front Oncol. 2020 Jun 24;10:906. doi: 10.3389/fonc.2020.00906 (PMC7327101; doi:10.3389/fonc.2020.00906)

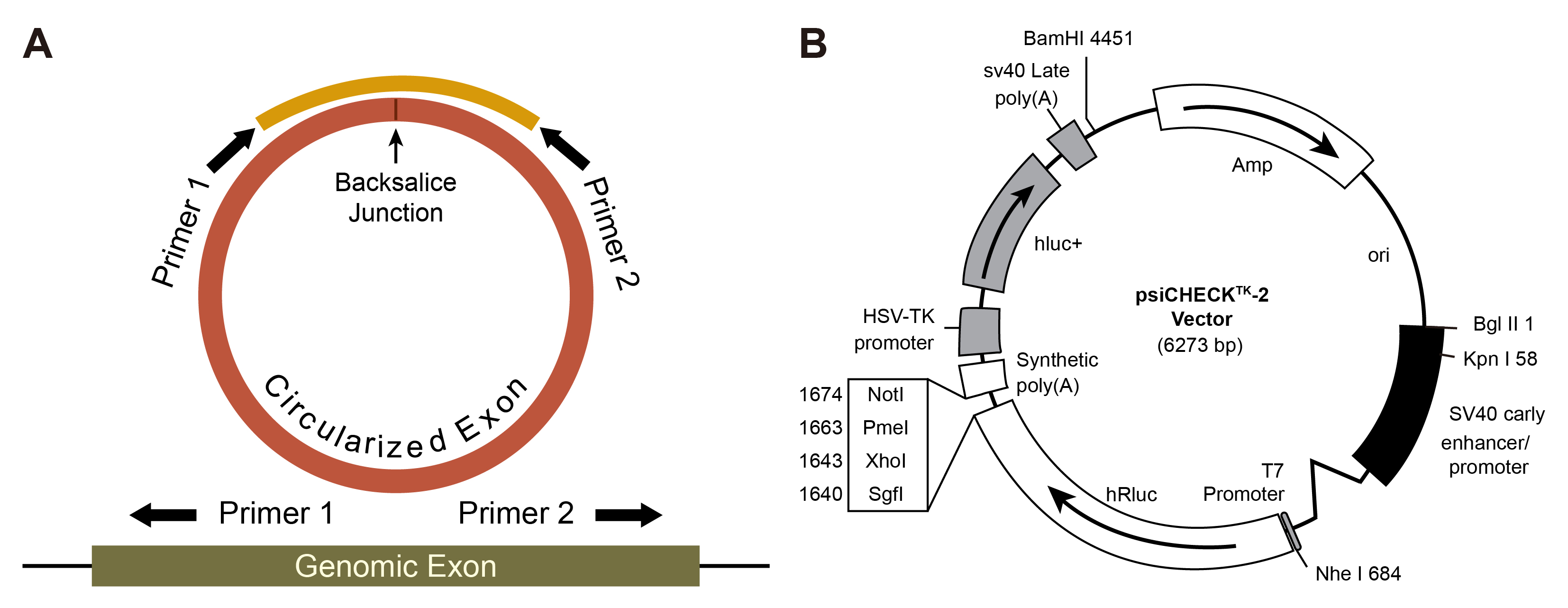

Supplement: Figure S1 — Schematic diagram of primers that distinguishing circRNA RNA and linear RNA (A) and plasmid structure of psiCHECK-2 (B). [file Image_1.JPEG]

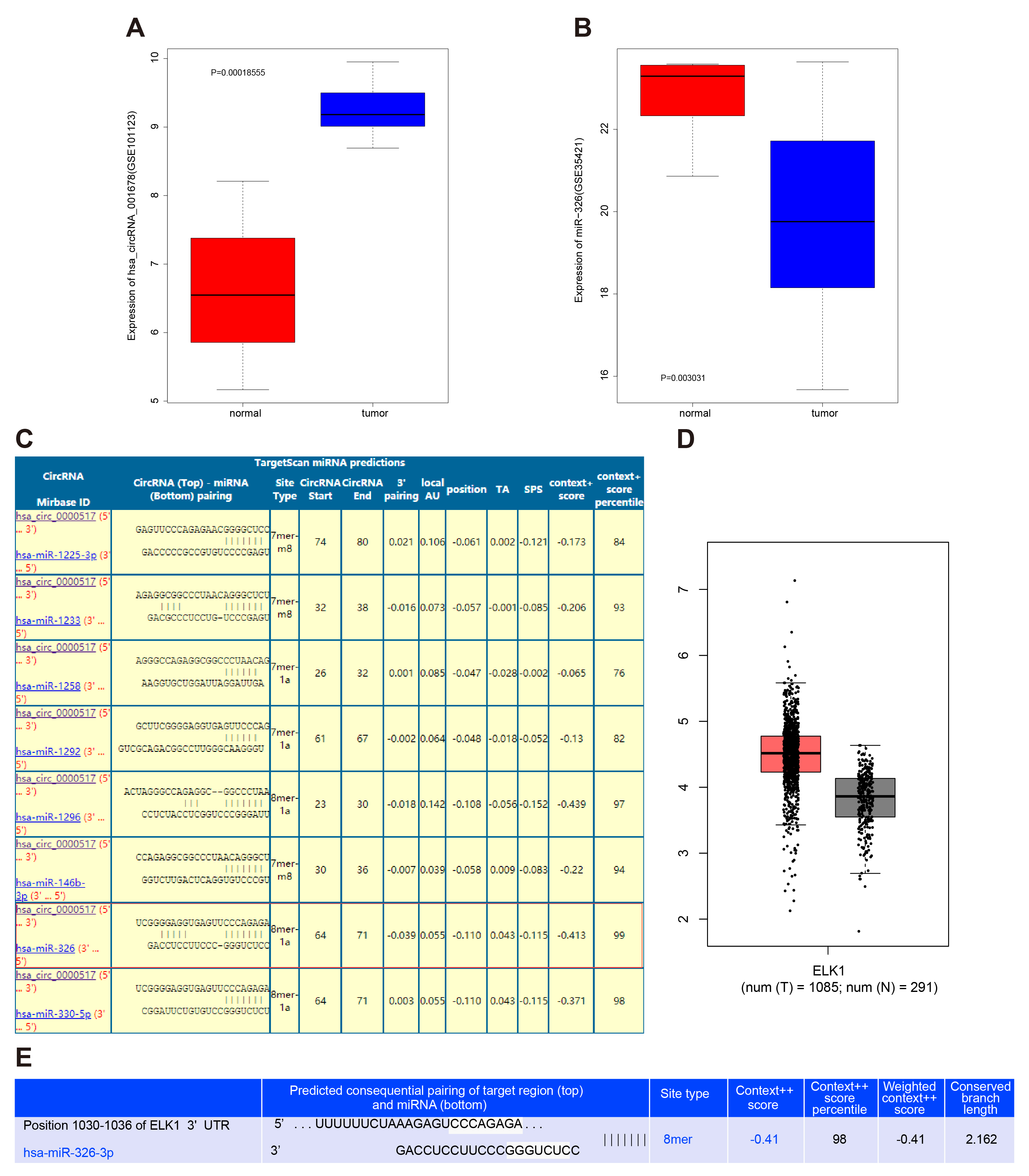

Supplement: Figure S2 — Bioinformatics revealed a possible regulatory mechanism involving hsa_circRPPH1_015 in BC. (A) The expression of hsa_circRNA_001678 in the microarray GSE110123. (B) The expression of miR-326 in tumor tissues and adjacent normal tissues in BC. (C) Prediction of the target miRNA of hsa_circRNA_001678 (hsa_circ_0000517) using CircInteractome database. (D) ELK1 expression in adjacent normal and tumor tissues in TCGA database, the abscissa represents the disease name and samples, the ordinate represents the TPM value, the red box graph represents the tumor sample, and the gray box graph represents the normal sample. (E) Binding sites between miR-326 and ELK1 predicted by the TargetScan database. BC, breast cancer; ELK1, ETS-domain containing protein; TCGA, the Cancer Genome Atlas; TPM, transcripts per million. [file Image_2.JPEG]

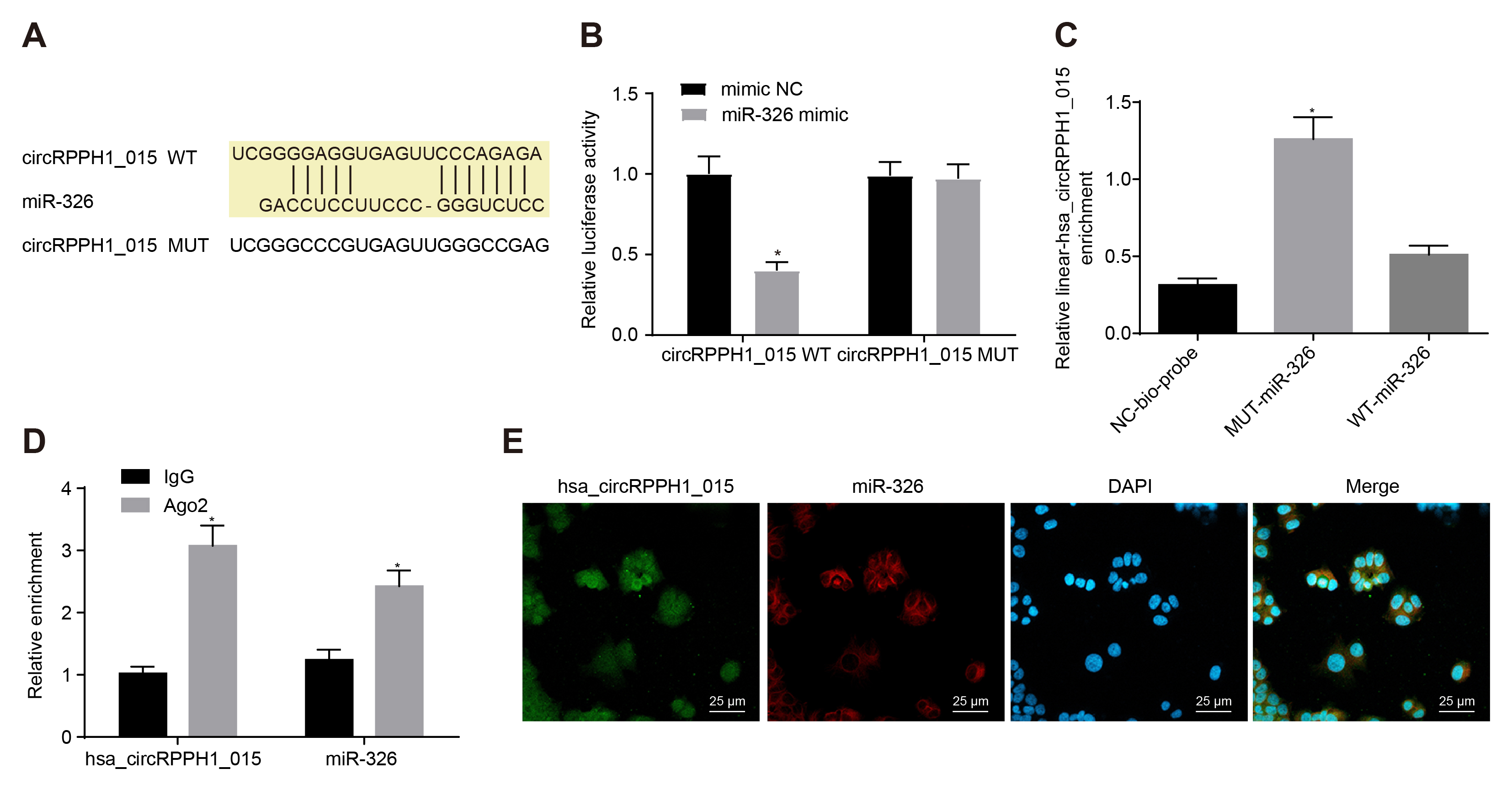

Supplement: Figure S3 — The miR-326 expression was inhibited by hsa_circRPPH1_015. (A) Prediction of the binding between hsa_circRPPH1_015 and miR-326. (B) Verification of the binding between hsa_circRPPH1_015 and miR-326 by dual-luciferase reporter gene assay in MDA-MB-435 cells, *p < 0.05 vs. the si-NC group (MDA-MB-435 cells transfected with si-NC). (C) The linear-hsa_circRPPH1_015 enrichment relative to NC-bio-probe detected by RNA pull-down experiment in MDA-MB-435 cells, *p < 0.05 vs. the NC-bio-probe group (MDA-MB-435 cells treated with NC-bio-probe). (D) The enrichment of Ago2 relative to IgG detected by RIP experiment in MDA-MB-435 cells, *p < 0.05 vs. the IgG group (MDA-MB-435 cells treated with IgG). (E) The cellular localization of hsa_circRPPH1_015 and miR-326 tested by FISH in MDA-MB-435 cells. The quantitative analysis results were measurement data and compared by paired or unpaired t-test between two groups and by one-way ANOVA among multiple groups. Values were obtained from three independent experiments. si-NC, small interfering RNA-negative control; RIP, RNA binding protein immunoprecipitation; FISH, fluorescence in situ hybridization; IgG, immunoglobulin G; ANOVA, analysis of variance. [file Image_3.JPEG]

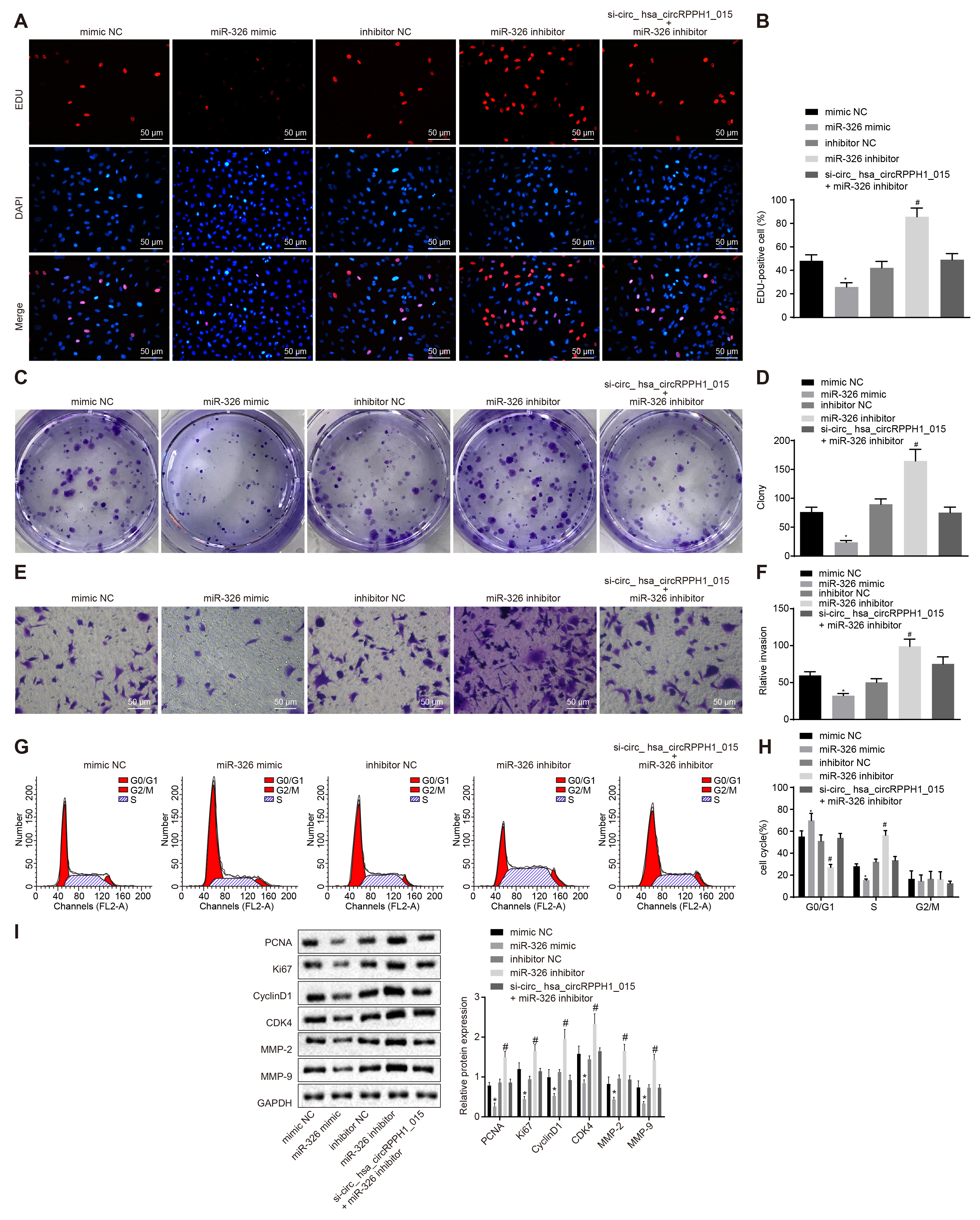

Supplement: Figure S4 — miR-326 inhibited the occurrence and development of BC. (A,B) MDA-MB-435 cell positive staining in each group examined by EdU assay. (C,D) Representative images of MDA-MB-435 colony formation and the quantification diagram examined by colony formation assay. (E,F) The invasion ability of MDA-MB-435 cells in each group examined by Transwell assay. (G,H) The cell cycle distribution of MDA-MB-435 cells in each group examined by flow cytometry. (I) The relative protein expression of associated proteins normalized to GAPDH in each group determined by Western blot analysis in MDA-MB-435 cells. *p < 0.05 vs. the mimic-NC group (MDA-MB-435 cells transfected with mimic-NC). #p < 0.05 vs. the inhibitor-NC group (MDA-MB-435 cells transfected with inhibitor-NC). The quantitative analysis results were measurement data and analyzed by one-way ANOVA among multiple groups. Values were obtained from three independent experiments. BC, breast cancer; EdU, 5-ethynyl-2′-deoxyuridine; GAPDH, glyceraldehyde-3-phosphate dehydrogenase; NC, negative control; ANOVA, analysis of variance. [file Image_4.JPEG]

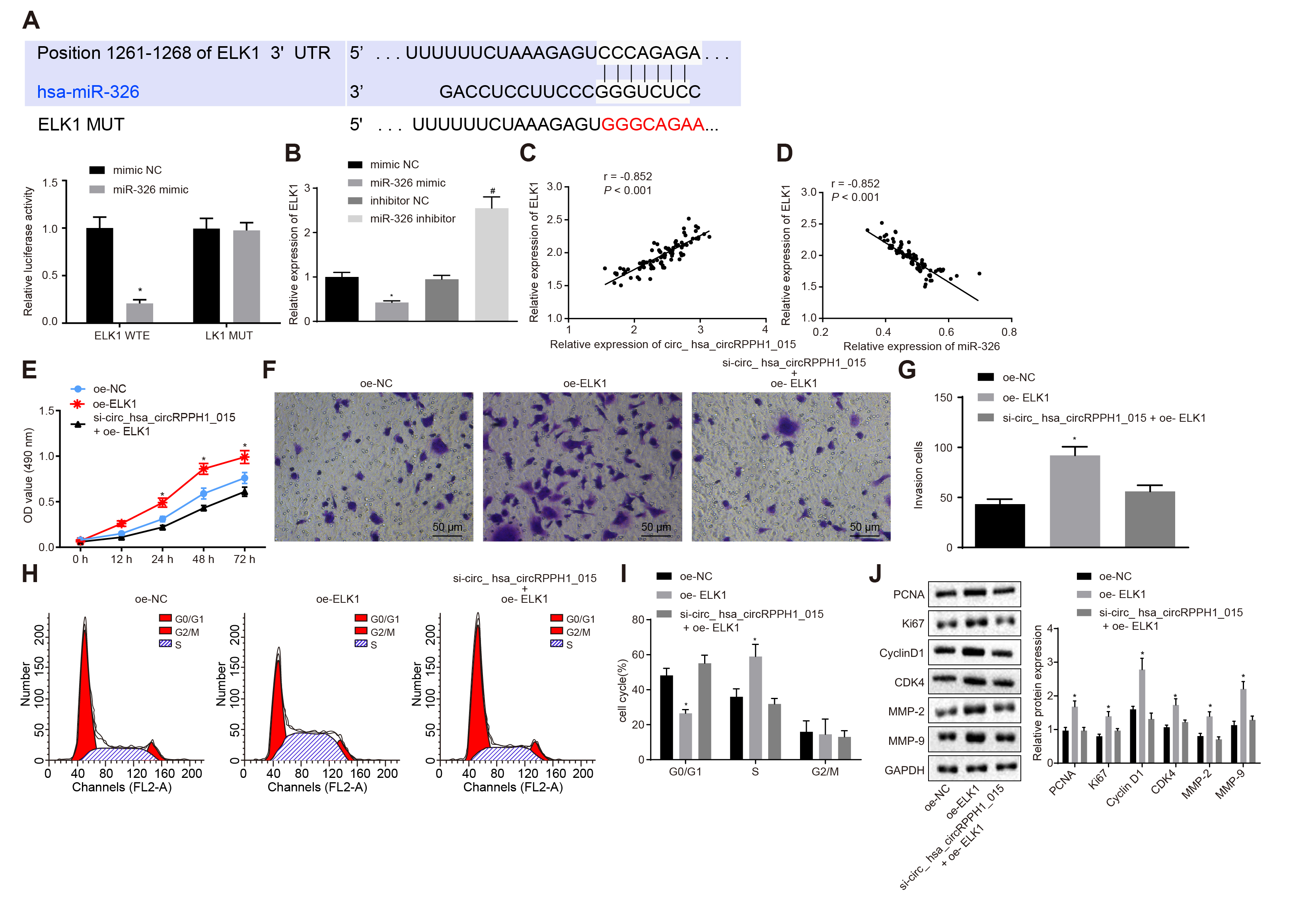

Supplement: Figure S5 — Regulation of ELK1 via miR-326 by hsa_circRPPH1_015 contributes to the development of BC. (A) The targeting relationship between miR-326 and ELK1 verified by dual-luciferase reporter gene assay in MDA-MB-435 cells. (B) The expression of ELK1 in MDA-MB-435 cells examined by RT-qPCR. (C) The correlation analysis between hsa_circRPPH1_015 and ELK1. (D) The correlation analysis between miR-326 and ELK1. (E) The proliferation of MDA-MB-435 cells in each group assessed by EdU assay. (F,G) The cell migration ability of MDA-MB-435 cells assessed by Transwell assay. (H,I) The distribution of MDA-MB-435 cell cycle in each group detected by flow cytometry. (J) The expression of associated proteins normalized to GAPDH in each group determined by Western blot analysis. *p < 0.05 vs. the mimic NC group (MDA-MB-435 cells transfected with mimic NC). #p < 0.05 vs. the inhibitor NC group (MDA-MB-435 cells transfected with inhibitor NC), the oe-NC group (MDA-MB-435cells transfected with oe-NC). The quantitative analysis results were measurement data and compared by unpaired t-test between two groups and by one-way ANOVA among multiple groups. Values were obtained from three independent experiments. BC, breast cancer; ELK1, ETS-domain containing protein; EdU, 5-ethynyl-2′-deoxyuridine; RT-qPCR, reverse transcription quantitative polymerase chain reaction; GAPDH, glyceraldehyde-3-phosphate dehydrogenase; NC, negative control; oe, overexpression; ANOVA, analysis of variance. [file Image_5.JPEG]
