# Supplementary material for: A Novel Circular RNA hsa_circRPPH1_015 Exerts an Oncogenic Role in Breast Cancer by Impairing miRNA-326-Mediated ELK1 Inhibition
Source: Front Oncol. 2020 Jun 24;10:906. doi: 10.3389/fonc.2020.00906 (PMC7327101; doi:10.3389/fonc.2020.00906)
Supplement: Supplementary Table 1 — Predicted target genes of miR-326. [file Table_1.docx]

**Supplementary table 1** Predicted target genes of miR-326

| TLN1 |
| --- |
| DRD2 |
| VLDLR |
| LRRTM1 |
| PALM |
| ELK1 |
| ANKFY1 |
| RALGPS2 |
| PAX8 |
| C9orf24 |
| KCNIP2 |
